# High intelligence is not associated with a greater propensity for mental health disorders

**DOI:** 10.1192/j.eurpsy.2022.2343

**Published:** 2022-11-18

**Authors:** Camille Michèle Williams, Hugo Peyre, Ghislaine Labouret, Judicael Fassaya, Adoración Guzmán García, Nicolas Gauvrit, Franck Ramus

**Affiliations:** 1 Laboratoire de Sciences Cognitives et Psycholinguistique, Département d’Études Cognitives, École Normale Supérieure, EHESS, CNRS, PSL University, 75005 Paris, France; 2 INSERM UMR 1141, Paris Diderot University, Paris, France; 3 Department of Child and Adolescent Psychiatry, Robert Debré Hospital, APHP, Paris, France; 4 Human and Artificial Cognition Lab, Ecole Pratique des Hautes Etudes, Paris, France

**Keywords:** Allergies, anxiety, intelligence, post-traumatic stress disorder, psychopathology

## Abstract

**Background:**

Studies reporting that highly intelligent individuals have more mental health disorders often have sampling bias, no or inadequate control groups, or insufficient sample size. We addressed these caveats by examining the difference in the prevalence of mental health disorders between individuals with high and average general intelligence (*g*-factor) in the UK Biobank.

**Methods:**

Participants with *g*-factor scores standardized relative to the same-age UK population, were divided into two groups: a high *g*-factor group (*g*-factor 2 SD above the UK mean; *N* = 16,137) and an average *g*-factor group (*g*-factor within 2 SD of the UK mean; *N* = 236,273). Using self-report questionnaires and medical diagnoses, we examined group differences in the prevalence of 32 phenotypes, including mental health disorders, trauma, allergies, and other traits.

**Results:**

High and average *g*-factor groups differed across 15/32 phenotypes and did not depend on sex and/or age. Individuals with high *g*-factors had less general anxiety (odds ratio [OR] = 0.69, 95% CI [0.64;0.74]) and post-traumatic stress disorder (PTSD; OR = 0.67, 95 %CI [0.61;0.74]), were less neurotic (β = −0.12, 95% CI [−0.15;−0.10]), less socially isolated (OR = 0.85, 95% CI [0.80;0.90]), and were less likely to have experienced childhood stressors and abuse, adulthood stressors, or catastrophic trauma (OR = 0.69–0.90). However, they generally had more allergies (e.g., eczema; OR = 1.13–1.33).

**Conclusions:**

The present study provides robust evidence that highly intelligent individuals do not have more mental health disorders than the average population. High intelligence even appears as a protective factor for general anxiety and PTSD.

## Introduction

Intelligence—the ability to learn, reason, and solve problems [1]—is associated with greater physical health and longevity. And yet, some researchers report that highly intelligent individuals are at a higher risk of developing mental health and somatic disorders [2–5]. The majority of studies that report a negative effect of intelligence on mental and somatic health, however, often suffer from sampling bias, the lack of a control group, or insufficient sample size [6, 7]. The present study addresses these caveats by examining the difference in the prevalence of mental health and somatic disorders and other traits between the highly intelligent (2 SD above mean) and averagely intelligent (within 2 SD of the mean) individuals of the UK Biobank (*N* ≃ 261,500).

The notion of general intelligence (*g*) stems from the positive correlation in performance across most cognitive tests [8]. It is formally defined as the common source of variance underlying performance across a wide range of tests and is usually computed using factor analysis. Intelligence quotient (IQ) reflects a person’s average performance across cognitive tests relative to a representative sample of the same-age national population. Henceforth, IQ and *g* will be used interchangeably for general intelligence (*g*-factor) scores.

Over the last decades, intelligence has proved to be a strong predictor of education [9], socioeconomic success [10, 11], and health outcomes [12–15]. For instance, having a childhood IQ one standard deviation (SD) above the mean decreases one’s risk of accidents and developing heart, respiratory, and digestive disease by 20–25% [14, 15]. And yet, whether intelligence serves as a protective factor for mental health disorders is still subject to debate. Whereas some postulate that high intelligence serves as a protective factor for several mental and somatic disorders[16–19], others suggest that high intelligence is a risk factor for these phenotypes [2, 4, 5, 20–22] or that the effects of intelligence vary across mental health phenotypes [23].

The most recent study examining the prevalence of mental health and somatic (i.e., allergies, asthma, and immunodeficiencies) disorders in highly intelligent individuals reported that high IQ was a risk factor for affective disorders, neurodevelopmental disorders, and diseases related to the immune system [2]. However, the study suffers from sampling bias because participants were recruited from the American Mensa Ltd.—a society open to individuals that at some point scored in the top 2% on a verified intelligence test (*N* = 3,715). Since IQ tests are typically administered to children when parents or teachers notice behavioral problems or by individuals experiencing stereotypical characteristics associated with IQ, selecting individuals from a sample of individuals who actively decided to take an IQ test or become members of a highly intelligent society may exacerbate the correlation between having a high IQ and mental health disorders and/or behavioral problems [6, 7]. The present study thus aims to address these limitations.

We investigated the difference in prevalence between individuals with high (2 SD above the population mean) and average (within 2 SD from the population mean) *g*-factor scores [24] in the UK Biobank across mental health disorders, somatic disorders, and certain traits. We examined group differences in the prevalence of available mental health and somatic disorders in the UK Biobank, as well as phenotypes that are thought to differ in prevalence in highly intelligent individuals, such as subjective well-being phenotypes (e.g., well-being and social isolation; [25, 26]), myopia [27], chronotype [28], and trauma [29]. Finally, we included sexual behaviors because studies have shown heterogeneous results concerning the relationship between sexual behaviors and IQ [30, 31]. As a point of comparison, we report differences in prevalence across phenotypes between the average and low (2 SD under the population mean) *g*-factor groups as exploratory analyses.

## Methods

Analyses were conducted using R [32]. R Packages are listed in Section S3 of the Supplementary Material and the preregistration, code, and supplementary materials are available at: https://osf.io/cywd6/?view_only=fa9f5091de124d96be3eb1a55a4e7f01.

### Participants

Participants were taken from the UK Biobank, an open-access large prospective study with phenotypic, genotypic, and neuroimaging data from more than 500,000 participants recruited between 2006 and 2011 at 40–69 years old [33]. All participants provided informed consent (“Resources tab” at https://biobank.ctsu.ox.ac.uk/crystal/field.cgi?id=200). All procedures involving human subjects/patients were approved by the Research Ethics Committee (reference 11/NW/0382). The present study was conducted based on the UK Biobank application 46007.

### G-factor

We defined our *g*-factor groups using *g*-factor scores from a previous study that are relative to the UK population [24]. In brief, we calculated *g*-factor scores for individuals that completed at least one of the UK Biobank cognitive tests.

UK Biobank participants completed a variety of cognitive tests upon each of their visits to the UK Biobank assessment center (Category 100026) and online (Category 116), including: Fluid Intelligence, Matrix Pattern Completion, Tower Rearranging, Numeric Memory, Pairs Matching, Symbol Digit Substitution, Reaction Time, and Trail Making tests (Section S1.1 of the Supplementary Material). Most tests were completed at the first visit and we used the score of the first instance of completion if the test was completed several times.

Although the UK Biobank cognitive battery contains adapted versions of commonly used tests and tests designed for the UK Biobank, the *g*-factor from well-validated tests correlates highly with the *g*-factor from the UK Biobank cognitive tests (*r* = 0.83; [34]).

Because UK Biobank participants tend to live in less socioeconomically deprived areas and are healthier than the UK population [35], we expected more than 2% of UK participants in the highly intelligent group (aka individuals with *g*-factor > 2 SD from the UK mean). To accurately identify highly intelligent individuals in the UK Biobank, we created a *g*-factor that would be relative to the UK mean.

To adjust for sampling bias, we calculated weights for each UK Biobank participant based on their country of residence, sex, age range, occupation status, and occupation SOC group using public the 2001 UK census data. We then adjusted for age with a semiparametric continuous norming method that considers weights [36].

From the age-standardized cognitive tests that are relative to the UK population, we extracted a *g*-factor from a single-factor model. We then computed the *g*-factor scores’ weighted mean and SD, using the census weighting factors. Finally, we standardized each person’s *g*-factor score with the weighted sample mean and weighted sample SD for a general population mean of 0 and SD of 1.

We estimated the quality of the *g*-factor based on the combination of completed tests, and we selected participants who had a reliable estimate of the *g*-factor (correlation with full *g-*factor > 0.7; *N* = 261,701 participants; Section S1.2 of the Supplementary Material). Participants were excluded when there was a mismatch between the self-reported (field 31) and genetic sex (field 22001). Self-reported sex was coded −0.5 for men and 0.5 for women.

### G-factor groups

We created three *g*-factor groups: a high *g*-factor group (*g*-factor 2 SD above the population mean), a low *g*-factor group (*g*-factor 2 SD below the population mean), and an average *g*-factor group (*g*-factor within 2 SD from the population mean). About 90% of individuals were in the average *g*-factor group (236,273/261,701), 6.2% in the high *g*-factor group (16,137/261,701), and 3.6% in the low *g*-factor group (9,291/261,701; sex differences in Section S1.2 of the Supplementary Material).

### Phenotypes

To maximize the number of participants with a diagnosis, each phenotype was created by combining questions and diagnoses from mental health professionals and self-reported questionnaires ([37–39]; Supplementary Table SA and Section S1.3 of the Supplementary Material). We examined 32 phenotypes using one or several binary, ordinal, or continuous variables (Supplementary Table SA, and Supplementary Tables SB1 and SB2).

Since the age of onset of disorders was not available for all participants and/or measures, we used the maximum age at which an individual provided the most recent measure of a disorder to approximate lifetime prevalence (Section S1.4 of the Supplementary Material).

**Table 1.** Phenotypic prevalence in the UK Biobank across and by sexes.

	Both sexes			Men			Women		
	Without	With		Without	With		Without	With	
Phenotypes	*N*	*N*	%	*N*	*N*	%	*N*	*N*	%
Eating disorder	124,092	1,489	1.19	54,488	116	0.21	69,604	1,373	1.93
Anorexia	124,886	750	0.6	54,583	35	0.06	70,303	715	1.01
Anxiety	69,555	12,337	15.06	34,437	4,219	10.91	35,118	8,118	18.78
Social anxiety	117,099	1,412	1.19	50,835	654	1.27	66,264	758	1.13
Depression ever	22,872	11,556	33.57	11,678	3,765	24.38	11,194	7,791	41.04
Probable depression	86,282	30,995	26.43	43,254	10,970	20.23	43,028	20,025	31.76
Bipolar disorder	30,035	226	0.75	14,494	108	0.74	15,541	118	0.75
Schizophrenia	125,387	194	0.15	54,497	107	0.2	70,890	87	0.12
Self-harm thought or action	220,343	41,079	15.71	103,443	15,215	12.82	116,900	25,864	18.12
Post-traumatic stress disorder	117,290	7,820	6.25	51,826	2,626	4.82	65,464	5,194	7.35
Obsessive–compulsive disorder	156,192	1,268	0.81	67,781	400	0.59	88,411	606	0.68
Alcohol hazardous substance use or dependence	117,083	6,591	5.33	49,594	4,072	7.59	67,489	2,519	3.6
Other hazardous substance use or dependence	258,625	2,760	1.06	117,119	1,520	1.28	141,506	1,240	0.87
Cannabis use ever	97,040	28,375	22.62	40,330	14,195	26.03	56,710	14,180	20
General allergy	73,287	29,550	28.73	32,990	12,538	27.54	40,297	17,012	29.68
Asthma	86,918	15,919	15.48	38,860	6,668	14.65	48,058	9,251	16.14
Hay fever rhinitis	450,094	51,200	10.21	205,755	22,680	9.93	244,339	28,520	10.45
Other allergies	489,588	11,682	2.33	224,543	3,880	1.7	265,045	7,802	2.86
Myopia	76,190	33,881	30.78	34,734	15,489	30.8	41,456	18,392	30.7
Eczema	252,947	8,448	3.23	114,743	3,902	3.29	138,204	4,546	3.18
Insomnia	57,376	204,142	78.06	32,753	85,948	72.41	24,623	118,194	82.76
Loneliness	452,782	23,105	4.86	204,356	11,782	5.45	248,426	11,323	4.36
Social isolation	444,466	45,053	9.2	201,014	21,856	9.81	243,452	23,197	8.7
Chronotype (more evening)	145,500	87,667	37.6	63,275	39,521	38.45	82,225	48,146	36.93
Same-sex sexual behavior	15,757	432,740	96.49	8,873	197,539	95.7	6,884	235,201	97.16
Adulthood abuse	20,479	20,479	16.32	4,701	4,701	8.62	15,778	15,778	22.25
Adulthood stressors	51,167	51,167	40.91	21,289	21,289	39.12	29,878	29,878	42.28
Catastrophic trauma	63,893	63,893	50.88	30,748	30,748	56.31	33,145	33,145	46.7
Childhood abuse	14,060	14,060	11.2	5,387	5,387	9.87	8,673	8,673	12.22
Childhood stressors	42,779	42,779	34.07	17,770	17,770	32.54	25,009	25,009	35.24

*Note.* Probable depression and cannabis use are ordinal but here reflect the prevalence of ever meeting the criteria regardless of severity.

### Statistical analyses

To reduce the number of statistical tests when examining group differences across phenotypes, we first examined age and sex effects and interactions on each phenotype (Section S1.5.1 of the Supplementary Material). If age and sex’s main effects or interactions did not predict a phenotype (*p* > 0.05), they were excluded from the *g*-factor group analyses. Equation 1 corresponds to the model with all possible predictors (*f* corresponds to the logit function for binary and ordinal phenotypes and to the linear function for continuous phenotypes).

Participants were included in the *g*-factor group analyses if they had a *g*-factor measure and answered all of the questions used to create that phenotype, except when the same question was asked several times. If participants responded as having the phenotype across any of the questions, they were marked as having the phenotype, otherwise, they were marked as not having the phenotype.

Age was mean-centered in ordinal and binary regressions, whereas age and continuous phenotypes were mean-centered and divided by 1 SD in linear regressions to report standardized betas.

***Equation 1:***


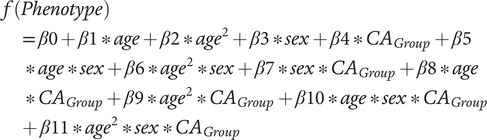



We report results with the *g*-factor group predictor that survived a Bonferroni correction for multiple comparisons of 0.05 divided by the number of coefficients in the equation with the *g*-factor group term times the number of coefficients of interest times the number of investigated phenotypes (e.g., 0.05/[6*32] for equation 1). Since the analyses on the low *g*-factor group were exploratory, we applied the same multiple comparison correction as for the high *g*-factor group (e.g., 0.05/[6*32] for equation 1). Odds ratio (OR) reflect the odds that a person will have a phenotype or disorder given that they are highly compared to averagely intelligent. ORs were calculated as the exponent of the regression estimate.

## Results

The model assumptions of low-powered phenotypes with few cases in the UK Biobank (including, anorexia, eating disorder, schizophrenia, bipolar disorder, obsessive–compulsive disorder [OCD], social anxiety, and other hazardous substance use) were not met and should be interpreted with caution (Supplementary Files S9–S40). We additionally ran the analyses with the *g*-factor scores instead of the *g*-factor group and found consistent results with our group analyses (Section S2.4 of the Supplementary Material, Supplementary Figures S5–S8, and Supplementary Files S9–S40).

### Differences in the prevalence of phenotypes between participants with high and average CA

Sex and age effects across phenotypes are described in Section S2.1 of the Supplementary Material and in Supplementary Table SC. In Supplementary Table SD, we provide the phenotypic prevalence by *g*-factor group and the mean and SD of the *g*-factor, Townsend Index, birth year, number of children, and number of individuals in the household of each *g*-factor group by diagnosis to describe the investigated populations. Regression results are reported in Supplementary Table SE.

**Figure 1.**
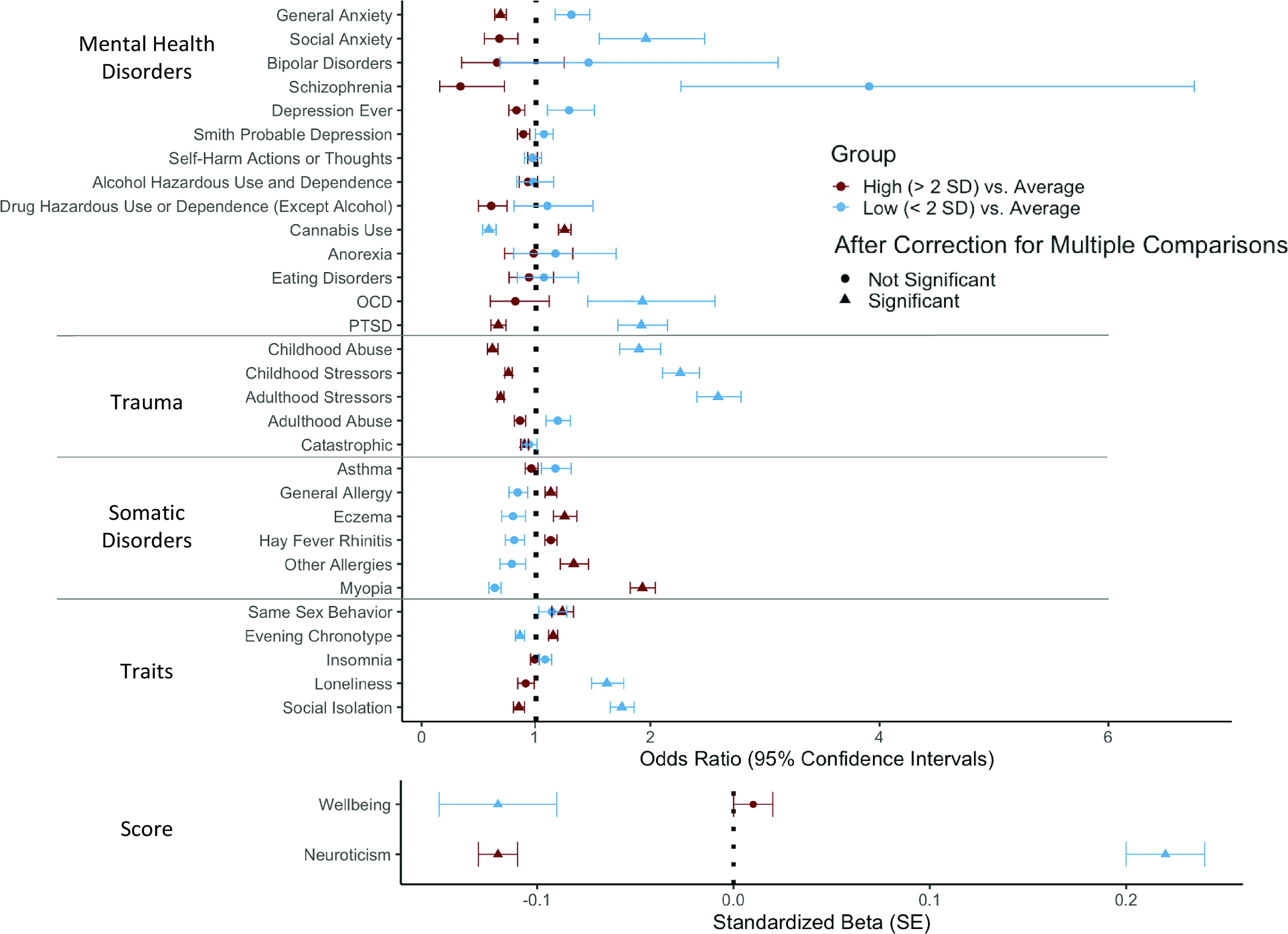
Group differences in prevalence between high and average and low and average *g*-factor Groups across phenotypes and scores. OCD, obsessive–compulsive disorder; PTSD, post-traumatic stress disorder. Correction for multiple comparisons varies by phenotype. See Supplementary tables standard error (SE) for *p*-value thresholds for multiple comparison corrections. High *g*-factor, participants with a *g*-factor score 2SD above the mean; low *g*-factor, participants with a *g*-factor score 2SD under the mean; average *g*-factor, participants with a *g*-factor score between ±2 SD from the mean.

Across the 32 phenotypes, the prevalence differed between the high *g*-factor and average *g*-factor groups in 15 phenotypes (47%) and between the low *g*-factor and average *g*-factor groups in 12 phenotypes (38%; Figure 1). Phenotypic differences between the low and average *g*-factor are discussed in Section S2.3 of the Supplementary Material. There were no significant interactions in the analyses comparing the average to the high *g*-factor groups.

#### Mental health disorders

Compared to individuals in the average *g*-factor group, there was a 33% decrease in the odds of suffering from post-traumatic stress disorder (OR = 0.67, 95% CI [0.61;0.74]) and a 31% decrease in the odds of having general anxiety (OR = 0.69, 95% CI [0.64;0.74]) in the high *g*-factor group (Table 2). There was no significant difference across other mental health disorders (Figure 1).

**Table 2.** Phenotypes that differ in prevalence between average and high general intelligence groups.

							Average *g*-factor			High *g*-factor		
		Estimate	SE	OR	*t*/*z*	*p*	Control	Cases	%	Control	Cases	%
High > Average	Other allergies	0.28	0.06	1.33	4.50	6.71E-06	229,995	6,012	2.55	15627	500	3.10
	Eczema	0.22	0.06	1.25	3.96	7.61E-05	228,451	7,556	3.20	15472	655	4.06
	Cannabis use ever	0.22	0.03	1.25	7.06	1.64E-12	86,279	24,836	22.35	8092	3,088	27.62
	Same-sex behavior	0.21	0.05	1.23	3.89	1.02E-04	209,239	8,741	4.01	14659	752	4.88
	Chronotype (E vs. M)	0.14	0.02	1.15	5.99	2.04E-09	132,775	82,172	38.23	8483	6,092	41.80
	General allergy	0.12	0.03	1.13	4.00	6.29E-05	64,788	25,833	28.51	6851	3,149	31.49
	Myopia	0.66	0.10	1.93	6.49	8.79E-11	70,191	30,851	30.53	2903	2,281	44.00
High < Average	Trauma catastrophic	−0.10	0.03	0.90	−3.70	2.20E-04	54,472	56,797	51.04	5640	5,554	49.62
	Social isolation	−0.16	0.04	0.85	−4.28	1.83E-05	210,478	23,110	9.89	14634	1,414	8.81
	Trauma childhood stressors	−0.27	0.03	0.76	−8.92	4.62E-19	73,178	38,076	34.22	8141	3,053	27.27
	Trauma adulthood stressors	−0.37	0.03	0.69	−12.43	1.83E-35	65,178	45,654	41.19	7608	3,565	31.91
	General anxiety	−0.37	0.05	0.69	−7.18	7.00E-13	61,127	11,118	15.39	6925	847	10.90
	Post-traumatic stress disorder	−0.40	0.07	0.67	−5.77	7.70E-09	103,826	7,022	6.33	10730	441	3.95
	Trauma childhood abuse	−0.48	0.05	0.62	−9.18	4.14E-20	98,544	12,688	11.41	10374	819	7.32
	Neuroticism score	−0.12	0.01		−10.17	2.87E-24						

*Note.* Neuroticism estimate is a standardized beta.

*Abbreviations:* *g*-factor, general intelligence; high *g*-factor, participants with a *g*-factor score 2 SD above the mean; average *g*-factor, participants with a *g*-factor score between ±2 SD from the mean; Cannabis use, never used versus used at least once; E, evening; M, morning.

#### Somatic phenotypes

Compared to individuals in the average *g*-factor group, the odds of having some type of allergy increased by 13% for individuals in the high *g*-factor group (OR = 1.13, 95% CI [1.08;1.18]). This was explained by their greater propensity to having eczema and other allergies (e.g., food; respectively, 1.25 and 1.33 times more likely), which were included in the general allergy diagnosis (Table 2). The odds of being myopic increased by 93% in the high *g*-factor group (OR = 1.93, 95% CI [1.82;2.04]) and this remained significant when controlling for educational attainment (OR = 1.75; Section S2.2 of the Supplementary Material).

#### Trauma

Compared to individuals in the average *g*-factor group, the odds of experiencing catastrophic trauma, adulthood stressors, childhood abuse, and childhood stressors decreased by 10% (OR = 0.90, 95% CI [0.87;0.94]), 31% (OR = 0.69, 95% CI [0.66;0.72]), 38% (OR = 0.62, 95% CI [0.58;0.67]), and 24% (OR = 0.76, 95% CI [0.73;0.79]; Table 2) in the high *g*-factor group, respectively.

#### Traits

Compared to individuals in the average *g*-factor group, the odds of feeling more socially isolated decreased by 15% in the high *g*-factor group (OR = 0.85, 95% CI [0.80;0.90]), whereas the odds of having an evening-like chronotype, ever engaging in same-sex behavior, and ever using cannabis increased by 15% (OR = 1.15, 95% CI [1.11;1.19]), 23% (OR = 1.23, 95% CI [1.14;1.33]), and 25% (OR = 1.25, 95% CI [1.20;1.31]), respectively, in the high *g*-factor group. Supplementary Figure S4 shows that there are more individuals with a higher-than-average *g*-factor that have used cannabis 1 to 100 times and that over 100 times there are no group differences. The high *g*-factor group had a lower neuroticism score than individuals in the average *g*-factor group (β = −0.12, SE = 0.01; Table 2).

### Phenotypes with group differences between both the high and average and the low and average g-factor groups

Childhood stressors, childhood abuse, adulthood stressors, PTSD, and social isolation were more prevalent in the low compared to the average *g*-factor group (Supplementary Table S2) and were more prevalent in the average compared to the high *g*-factor group (Table 2), suggesting that the prevalence of these phenotypes decreases with an increasing *g*-factor. The low *g*-factor group had a higher neuroticism score than the average *g*-factor group (β = 0.22, SE = 0.02), which had a higher neuroticism score than the high *g*-factor group. The odds of ever trying cannabis and having an evening-like chronotype, respectively, decreased by 41% (OR = 0.59, 95% CI [0.53;0.65]) and 14% (OR = 0.86, 95% CI [0.82;0.90]) in the low compared to the average *g*-factor group.

## Discussion

We examined differences in the prevalence of mental health disorders, somatic disorders, and certain traits between individuals with high (2 SD above mean) and average *g*-factor scores (within 2 SD of the mean) in the UK Biobank (*N* ≃ 7,266–252,249). We contrasted these results with differences observed between individuals with low and average *g*-factor scores.

We found that the high *g*-factor group did not have more mental health disorders than the average *g*-factor group and that they were less likely to have general anxiety and PTSD. Individuals with higher intelligence were also less likely to have experienced trauma and stressors, except for adulthood abuse, which may be part of the explanation for the latter finding. The high *g*-factor group was also less neurotic and felt less socially isolated than the average g-factor group. In contrast, the low *g*-factor group was more neurotic, felt more socially isolated, and had a greater prevalence of trauma, stressors, and PTSD than the average *g*-factor group, suggesting that the prevalence of these phenotypes decreases with increasing intelligence. Among the few somatic disorders that were examined, we found that individuals with high intelligence were more myopic and had more allergies, although they had a lower prevalence of hay fever rhinitis and asthma. Individuals with high intelligence were also more likely to present certain traits, such as having an afternoon–evening chronotype, to have ever tried cannabis, or have ever engaged in same-sex behavior, whereas the low *g*-factor group was less likely to have ever tried cannabis and engaged in same-sex behavior than the average *g*-factor group. There were no differences between groups in the prevalence of insomnia.

Our results contradicts several studies that reported an increased risk for various psychiatric disorders in individuals with high intelligence (e.g., [2, 5]). These studies were generally based on small samples and suffered from major sampling bias or a lack of a control group [6, 7]. We find that high intelligence is not a risk factor for psychiatric disorders and even a protective factor for general anxiety. Higher intelligence was associated with a decrease in trauma exposure, and consequently PTSD. This is consistent with previous findings [29] and with the association of childhood trauma with lower intelligence [40].

With regard to somatic disorders, we replicate the increased risk of allergies in individuals with high intelligence [2, 41]. One possible explanation for this association is that allergies and intelligence share neural correlates [42]. Another possibility is that more intelligent individuals with a higher *g*-factor live in more urban areas [43], where allergies are more prevalent [44], or that individuals with high intelligence are more aware of allergic symptoms and have better access to health care. However, the prevalence between groups did not differ across all allergies (e.g., asthma and hay fever rhinitis).

In line with a previous literature review [27], the risk of myopia was greater for individuals with high intelligence. While near-work activities (e.g., reading and computer use) seem to be a risk factor for myopia [45, 46], this association appears to be distinct from that of higher intelligence and education level [22]. Although additional years of education contribute to an increase in the risk of developing myopia [47], most of the evidence points towards shared genetic factors between intelligence and myopia [22], which is consistent with our observation that the risk of myopia associated with a high *g*-factor only slightly decreased when adjusting for educational attainment.

Our results indicate more afternoon–evening chronotypes in individuals with high intelligence than in individuals with average intelligence, which could be explained by differences in the work schedules of the different *g*-factor groups [28]. In line with a previous study [30], we find that individuals with high intelligence are more likely to ever have engaged in same-sex behavior. However, we note that our measure reflects sexual exploration rather than sexual orientation. We also found an association between ever trying cannabis and intelligence, but this was only true for individuals who consumed cannabis less than 101 times in their lifetime, suggesting that this measure also reflects a tendency to explore. One possibility is that individuals with higher intelligence, which is positively correlated with the “Openness to Experience” personality trait (*r* = 0.30; [49]), may be more likely to seek out new experiences and explore alternative behaviors than the average.

This study was first limited in its ability to study neurodevelopmental disorders and some psychotic disorders, which were absent or had few cases in the UK Biobank. We nonetheless investigated phenotypic differences in prevalence between *g*-factor groups across a greater number of psychiatric disorders than previously done [2, 50] and examined additional phenotypes, including traumatic experiences.

Second, the prevalence of some psychiatric disorders and traits differs from the general population [36] because of the UK Biobank’s “healthy volunteer” bias [35]. However, this should not affect the validity of the group comparisons because the phenotypes were designed with the same criterion across high and average g-factor groups.

Third, while some data may not be missing at random, our participants are more representative of highly intelligent individuals in the general population than those from previous Mensa studies, which did not have a control group and examined individuals that had to take or decided to take an IQ test and join a society for the highly intelligent.

Fourth, although we used self-report diagnoses, which are less precise than professional diagnoses, UK Biobank participants self-reporting any psychiatric diagnosis appear to have an elevated risk of any symptom-based outcome [51]. By using professional and probable diagnoses with validated short mental health tests, we were also able to include individuals with mental health disorders that have not sought or received mental health assistance [52, 53].

Fifth, the prevalence of psychiatric disorders and traits between *g*-factor groups may differ across the lifespan. However, we were interested in lifetime prevalence, making the UK Biobank, a prospective aging study, a good candidate for the question at hand.

Therefore, while this study is not without limitations, we provide a large scaled analysis of the difference in the prevalence of numerous phenotypes, including mental health and somatic disorders, across individuals of varying intelligence. We find that high intelligence is not a risk factor for psychiatric disorders and is a protective factor for general anxiety and PTSD. Whereas our results support the advantageous nature of higher intelligence [50], *the g*-factor may still be relevant for psychiatric evaluation: higher intelligence may affect the presentation of symptoms and the available resources for recovery.

## Data Availability

This research has been conducted using data from UK Biobank, a major biomedical database (http://www.ukbiobank.ac.uk/). Restrictions apply to the availability of these data, which were used under license for this study: application 46007. Preregistration, code, and Supplementary tables and data are available on OSF: https://osf.io/cywd6/?view_only=fa9f5091de124d96be3eb1a55a4e7f01. Preprint: https://doi.org/10.1101/2022.05.26.22275621.

## Supplementary material

For supplementary material accompanying this paper visit https://doi.org/10.1192/j.eurpsy.2022.2343. click here to view supplementary material

## References

[1] Arvey RD, Bouchard TJ, Carroll JB, Cattell RB, Cohen DB, Dawis RV, et al. Mainstream science on intelligence. Wall Street J. 1994;13(1):18–25.

[2] Karpinski RI, Kolb AMK, Tetreault NA, Borowski TB. High intelligence: a risk factor for psychological and physiological overexcitabilities. Intelligence. 2018;66:8–23. doi:10.1016/j.intell.2017.09.001.

[3] Kermarrec S, Attinger L, Guignard J-H, Tordjman S. Anxiety disorders in children with high intellectual potential. BJPsych Open. 2020;6(4). doi:10.1192/bjo.2019.104. e70. 32627729 PMC7443907

[4] Lancon C, Martinelli M, Michel P, Debals M, Auquier P, Guedj E, et al. Comorbidités psychiatriques et qualité de vie chez les sujets adultes à haut potentiel intellectuel: relations avec l’estime de soi. Presse Med. 2015;44(5):e177–84. doi:10.1016/j.lpm.2014.11.018. 25813095

[5] Smith DJ, Anderson J, Zammit S, Meyer TD, Pell JP, Mackay D. Childhood IQ and risk of bipolar disorder in adulthood: prospective birth cohort study. BJPsych Open. 2015;1(1):74–80. doi:10.1192/bjpo.bp.115.000455. 27703726 PMC4995557

[6] Gauvrit N. Précocité intellectuelle: Un champ de recherches miné. ANAE - Approche Neuropsychologique Des Apprentissages Chez l’Enfant. 2014;132/133:527–34.

[7] Martin LT, Burns RM, Schonlau M. Mental disorders among gifted and nongifted youth: a selected review of the epidemiologic literature. Gift Child Q. 2010;54(1):31–41. doi:10.1177/0016986209352684.

[8] Spearman C. General intelligence. Objectively determined and measured. Am J Psychol. 1904;15(2):201–92. doi:10.2307/1412107.

[9] Deary IJ, Strand S, Smith P, Fernandes C. Intelligence and educational achievement. Intelligence. 2007;35(1):13–21. doi:10.1016/j.intell.2006.02.001.

[10] Schmidt FL, Hunter J. General mental ability in the world of work: occupational attainment and job performance. J Pers Soc Psychol. 2004;86(1):162–73. doi:10.1037/0022-3514.86.1.162. 14717634

[11] Strenze T. Intelligence and socioeconomic success: a meta-analytic review of longitudinal research. Intelligence. 2007;35(5):401–26. doi:10.1016/j.intell.2006.09.004.

[12] Calvin CM, Batty GD, Der G, Brett CE, Taylor A, Pattie A, et al. Childhood intelligence in relation to major causes of death in 68 year follow-up: prospective population study. BMJ. 2017;357. doi:10.1136/bmj.j2708. PMC5485432 28659274

[13] Twig G, Tirosh A, Derazne E, Haklai Z, Goldberger N, Afek A, et al. Cognitive function in adolescence and the risk for premature diabetes and cardiovascular mortality in adulthood. Cardiovasc Diabetol. 2018;17(1):154. doi:10.1186/s12933-018-0798-5. 30518353 PMC6280532

[14] Wraw C, Der G, Gale CR, Deary IJ. Intelligence in youth and health behaviours in middle age. Intelligence. 2018;69:71–86. doi:10.1016/j.intell.2018.04.005. 30100645 PMC6075942

[15] Wrulich M, Brunner M, Stadler G, Schalke D, Keller U, Martin R. Forty years on: childhood intelligence predicts health in middle adulthood. Health Psychol. 2014;33(3):292–6. doi:10.1037/a0030727. 23205542

[16] Burdick KE, Gunawardane N, Woodberry K, Malhotra AK. The role of general intelligence as an intermediate phenotype for neuropsychiatric disorders. Cogn Neuropsychiatry. 2009;14(4–5):299–311. doi:10.1080/13546800902805347. 19634032 PMC2727853

[17] Ghaffari J, Abbaskhanian A, Jalili M, Yazdani Charati J. IQ score of children with persistent or perennial allergic rhinitis: a comparison with healthy children. Iran J Child Neurol. 2014;8(3):44–8. 25143773 PMC4135280

[18] Koenen KC, Moffitt TE, Roberts AL, Martin LT, Kubzansky L, Harrington H, et al. Childhood IQ and adult mental disorders: a test of the cognitive reserve hypothesis. Am J Psychiatr. 2009;166(1):50–7. doi:10.1176/appi.ajp.2008.08030343. 19047325 PMC2705657

[19] Sjölund S, Hemmingsson T, Allebeck P. IQ and level of alcohol consumption—Findings from a National Survey of Swedish conscripts. Alcohol Clin Exp Res. 2015;39(3):548–55. doi:10.1111/acer.12656. 25702705 PMC4368388

[20] Kanazawa S. Evolutionary psychology and intelligence research. Am Psychol. 2010;65(4):279–89. doi:10.1037/a0019378. 20455621

[21] White J, Batty GD. Intelligence across childhood in relation to illegal drug use in adulthood: 1970 British cohort study. J Epidemiol Community Health. 2012;66(9):767–74. doi:10.1136/jech-2011-200252. 22086967

[22] Williams KM, Hysi PG, Yonova-Doing E, Mahroo OA, Snieder H, Hammond CJ. Phenotypic and genotypic correlation between myopia and intelligence. Sci Rep. 2017;7(1):Article 1. doi:10.1038/srep45977. PMC5382686 28383074

[23] Wraw C, Deary IJ, Der G, Gale CR. Intelligence in youth and mental health at age 50. Intelligence. 2016;58:69–79. doi:10.1016/j.intell.2016.06.005. 27642201 PMC5014225

[24] Williams CM, Labouret G, Wolfram T, Peyre H, Ramus F. A general cognitive ability factor for the UK Biobank. Behav Genet (2022). 10.1007/s10519-022-1 36378351

[25] Li NP, Kanazawa S. Country roads, take me home… to my friends: how intelligence, population density, and friendship affect modern happiness. Br J Psychol. 2016;107(4):675–97. doi:10.1111/bjop.12181. 26847844

[26] Zettergren P, Bergman LR. Adolescents with high IQ and their adjustment in adolescence and midlife. Res Hum Dev. 2014;11(3):186–203. doi:10.1080/15427609.2014.936182.

[27] Czepita D, Lodygowska E, Czepita M. Are children with myopia more intelligent? A literature review. Ann Acad Med Stetin. 2008;54(1):13–16; discussion 16. 19127804

[28] Ujma PP, Baudson TG, Bódizs R, Dresler M. The relationship between chronotype and intelligence: the importance of work timing. Sci Rep. 2020;10(1):Article 1. doi:10.1038/s41598-020-62917-9. PMC7188801 32345987

[29] McGuire A, Jackson Y. The role of trauma type and age in the relation between trauma exposure and intelligence. Child Maltreat. 2020;25(2):192–202. doi:10.1177/1077559519860596. 31288552

[30] Rahman Q, Bhanot S, Emrith-Small H, Ghafoor S, Roberts S. Gender nonconformity, intelligence, and sexual orientation. Arch Sex Behav. 2012;41(3):623–30. doi:10.1007/s10508-011-9737-1. 21331499

[31] Rowe DC. IQ, birth weight, and number of sexual partners in White, African American, and mixed race adolescents. Popul Environ. 2002;23(6):513–24. doi:10.1023/A:1016313718644.

[32] R Core Team. R: a language and environment for statistical computing. R Foundation for Statistical Computing. https://www.R-project.org/; 2022.

[33] Sudlow C, Gallacher J, Allen N, Beral V, Burton P, Danesh J, et al. UK Biobank: an open access resource for identifying the causes of a wide range of complex diseases of middle and old age. PLoS Med. 2015;12(3):e1001779. doi:10.1371/journal.pmed.1001779. 25826379 PMC4380465

[34] Fawns-Ritchie C, Deary IJ. Reliability and validity of the UK Biobank cognitive tests. PLoS One. 2020;15(4):e0231627. doi:10.1371/journal.pone.0231627. 32310977 PMC7170235

[35] Fry A, Littlejohns TJ, Sudlow C, Doherty N, Adamska L, Sprosen T, et al. Comparison of sociodemographic and health-related characteristics of UK Biobank participants with those of the general population. Am J Epidemiol. 2017;186(9):1026–34. doi:10.1093/aje/kwx246. 28641372 PMC5860371

[36] Lenhard, A., Lenhard, W., Suggate, S. & Segerer, R. A Continuous solution to the norming problem. Assessment 25, 112–125 (2016). 27371826 10.1177/1073191116656437

[37] Davis KAS, Coleman JRI, Adams M, Allen N, Breen G, Cullen B, Dickens C, Fox E, Graham N, Holliday J, Howard LM, John A, Lee W, McCabe R, McIntosh A, Pearsall R, Smith DJ, Sudlow C, Ward J, Zammit S, Hotopf M. Mental health in UK Biobank—Development, implementation and results from an online questionnaire completed by 157 366 participants: a reanalysis. BJPsych Open. 2020;6(2): e18. doi:10.1192/bjo.2019.100. PMID: 32026800; PMCID: PMC7176892. 32026800 PMC7176892

[38] Guggenheim JA, Williams C, for the UK Biobank Eye and Vision Consortium. Role of educational exposure in the association between myopia and birth order. JAMA Ophthalmology. 2015;133(12):1408–14. doi:10.1001/jamaophthalmol.2015.3556. PMC4681114 26448589

[39] Smith DJ, Nicholl BI, Cullen B, Martin D, Ul-Haq Z, Evans J, et al. Prevalence and characteristics of probable major depression and bipolar disorder within UK Biobank: cross-sectional study of 172,751 participants. PLoS One. 2013;8(11):e75362. doi:10.1371/journal.pone.0075362. 24282498 PMC3839907

[40] Enlow MB, Egeland B, Blood EA, Wright RO, Wright RJ. Interpersonal trauma exposure and cognitive development in children to age 8 years: a longitudinal study. J Epidemiol Community Health. 2012;66(11):1005–10. doi:10.1136/jech-2011-200727. 22493459 PMC3731065

[41] Benbow CP. Intellectually gifted students also suffer from immune disorders. Behav Brain Sci. 1985;8(3):442–2. doi:10.1017/S0140525X00001059.

[42] Takeuchi H, Taki Y, Nouchi R, Yokoyama R, Kotozaki Y, Nakagawa S. Allergic tendencies are associated with larger gray matter volumes. Sci Rep. 2018;8(1):Article 1. doi:10.1038/s41598-018-21985-8. PMC5829247 29487315

[43] Jokela M. Flow of cognitive capital across rural and urban United States. Intelligence. 2014;46:47–53. doi:10.1016/j.intell.2014.05.003.

[44] Schröder PC, Li J, Wong GWK, Schaub B. The rural–urban enigma of allergy: what can we learn from studies around the world? Pediatr Allergy Immunol. 2015;26(2):95–102. doi:10.1111/pai.12341. 25620193

[45] Huang H-M, Chang DS-T, Wu P-C. The association between near work activities and myopia in children—A systematic review and meta-analysis. PLoS One. 2015;10(10):e0140419. doi:10.1371/journal.pone.0140419. 26485393 PMC4618477

[46] Mutti DO, Mitchell GL, Moeschberger ML, Jones LA, Zadnik K. Parental myopia, near work, school achievement, and children’s refractive error. Invest Ophthalmol Vis Sci. 2002;43(12):3633–40. 12454029

[47] Mountjoy E, Davies NM, Plotnikov D, Smith GD, Rodriguez S, Williams CE, et al. Education and myopia: assessing the direction of causality by mendelian randomisation. BMJ (Clinical Research Ed). 2018;361:k2022. doi:10.1136/bmj.k2022. PMC5987847 29875094

[48] Willmott M, Brierley H. Cognitive characteristics and homosexuality. Arch Sex Behav. 1984;13(4):311–9. doi:10.1007/BF01541904. 6487075

[49] Stankov L. Low correlations between intelligence and big five personality traits: need to broaden the domain of personality. J Intelligence. 2018;6(2):E26. doi:10.3390/jintelligence6020026. PMC6480733 31162453

[50] Brown MI, Wai J, Chabris CF. Can you ever be too smart for your own good? Comparing linear and nonlinear effects of cognitive ability on life outcomes. Perspect Psychol Sci. 2021;16(6):1337–59. doi:10.1177/1745691620964122. 33682520

[51] Davis KAS, Cullen B, Adams M, Brailean A, Breen G, Coleman JRI, et al. Indicators of mental disorders in UK Biobank-a comparison of approaches. Int J Methods Psychiatr Res. 2019;28(3):e1796. doi:10.1002/mpr.1796. 31397039 PMC6877131

[52] Kessler RC, Amminger GP, Aguilar-Gaxiola S, Alonso J, Lee S, Ustun TB. Age of onset of mental disorders: a review of recent literature. Curr Opin Psychiatry. 2007;20(4):359–64. doi:10.1097/YCO.0b013e32816ebc8c. 17551351 PMC1925038

[53] Newson RS, Karlsson H, Tiemeier H. Epidemiological fallacies of modern psychiatric research. Nord J Psychiatry. 2011;65(4):226–37. doi:10.3109/08039488.2010.539268. 21138402

