# High intelligence is not associated with a greater propensity for mental health disorders – CORRIGENDUM

**DOI:** 10.1192/j.eurpsy.2025.10077

**Published:** 2025-09-23

**Authors:** Camille Michèle Williams, Hugo Peyre, Ghislaine Labouret, Judicael Fassaya, Adoración Guzmán García, Nicolas Gauvrit, Franck Ramus

**Keywords:** Allergies, anxiety, intelligence, post-traumatic stress disorder, psychopathology, corrigendum

In this article, Table 1 was published with a number of errors in the values. The corrected Table 1 is below:

**Table 1:** 

	Both Sexes			Men			Women		
	without	with		without	with		without	with	
	*N*	*N*	%	*N*	*N*	%	*N*	*N*	%
Same Sex Behavior	432,740	15,757	3.51%	197,539	15,757	7.39%	235,201	6,884	2.84%
Adulthood Abuse	104,979	20,479	16.32%	49,856	4,701	8.62%	55,123	15,778	22.25%
Adulthood Stresstors	73,919	51,167	40.91%	33,125	21,289	39.12%	40,794	29,878	42.28%
Catastrophic	61,694	63,893	50.88%	23,858	30,748	56.31%	37,836	33,145	46.70%
Childhood Abuse	111,485	14,060	11.20%	49,207	5,387	9.87%	62,278	8,673	12.22%
Childhood Stresstors	82,793	42,779	34.07%	36,832	17,770	32.54%	45,961	25,009	35.24%

In addition, there is an error on page 6 of the article, which currently reads as follows:

Individuals with high intelligence were also more likely to present certain traits, such as having an afternoon–evening chronotype, to have ever tried cannabis, or have ever engaged in same-sex behavior, whereas the low g-factor group was less likely to have ever tried cannabis and engaged in same-sex behavior than the average g-factor group.

The correct paragraph should be as follows:

Individuals with high intelligence were also more likely to present certain traits, such as having an afternoon–evening chronotype, to have ever tried cannabis, or have ever engaged in same-sex behavior, whereas the low g-factor group was less likely to have ever tried cannabis than the average g-factor group.
