# Supplementary material for: High intelligence is not associated with a greater propensity for mental health disorders
Source: Eur Psychiatry. 2022 Nov 18;66(1):e3. doi: 10.1192/j.eurpsy.2022.2343 (PMC9879926; doi:10.1192/j.eurpsy.2022.2343)
Supplement: Supplementary file 1 [file S0924933822023434sup001.zip › S0924933822023434sup010.html]

Neuroticism


# Neuroticism

#### 2022-10-14

## 1. CA Group Regressions

```
## [1] "There are 214049 individuals without missing data in this analysis."
```

```
##                                              Std Beta   SE        p
## (Intercept)                                      0.01 0.00 3.88e-02
## CA_GroupHigh_CA                                 -0.12 0.01 2.87e-24
## CA_GroupLow_CA                                   0.22 0.02 1.65e-32
## Sex                                              0.28 0.01 0.00e+00
## scale(Age_Center_0)                             -0.10 0.00 0.00e+00
## I(scale(Age_Center_0)^2)                        -0.02 0.00 4.02e-11
## CA_GroupHigh_CA:Sex                             -0.05 0.02 4.44e-02
## CA_GroupLow_CA:Sex                              -0.09 0.04 1.09e-02
## CA_GroupHigh_CA:scale(Age_Center_0)             -0.02 0.01 6.71e-02
## CA_GroupLow_CA:scale(Age_Center_0)              -0.02 0.01 5.67e-02
## Sex:scale(Age_Center_0)                         -0.01 0.00 1.79e-01
## CA_GroupHigh_CA:I(scale(Age_Center_0)^2)        -0.01 0.01 2.12e-01
## CA_GroupLow_CA:I(scale(Age_Center_0)^2)         -0.01 0.01 5.03e-01
## Sex:I(scale(Age_Center_0)^2)                    -0.01 0.00 5.45e-02
## CA_GroupHigh_CA:Sex:scale(Age_Center_0)          0.01 0.02 5.75e-01
## CA_GroupLow_CA:Sex:scale(Age_Center_0)           0.05 0.02 4.82e-02
## CA_GroupHigh_CA:Sex:I(scale(Age_Center_0)^2)    -0.01 0.02 6.04e-01
## CA_GroupLow_CA:Sex:I(scale(Age_Center_0)^2)      0.06 0.03 1.25e-02
```

## 2. Regression with g-factor Group Assumptions

Please not that violations of assumptions are likely due to the
ordinal characteristic of the neuroticism score

### a) Linearity

If there is no pattern in the residual plot. This suggests that we
can assume linear relationship between the predictors and the outcome
variables.

### b) Homogeneity of variance

It’s good if you see a horizontal line with equally spread
points.

### c) Normality of residuals

The normal probability plot of residuals should approximately follow
a straight line.

### d) Outliers and high levarage points

If there is no outliers that exceed 3 standard deviations, it is
good.

### e) Influential values

A rule of thumb is that an observation has high influence if Cook’s
distance exceeds 4/(n - p - 1)(P. Bruce and Bruce 2017), where n is the
number of observations and p the number of predictor variables. The
Residuals vs Leverage plot can help us to find influential observations
if any. On this plot, outlying values are generally located at the upper
right corner or at the lower right corner. Those spots are the places
where data points can be influential against a regression line.

## 3. Regression with g-factor

```
## [1] "There are 214049 individuals without missing data in this analysis."
```

```
##                                    Std Beta   SE         p
## (Intercept)                            0.02 0.00  1.17e-11
## G_std                                 -0.07 0.00 1.51e-152
## Sex                                    0.27 0.01  0.00e+00
## scale(Age_Center_0)                   -0.11 0.00  0.00e+00
## I(scale(Age_Center_0)^2)              -0.02 0.00  4.49e-17
## G_std:Sex                             -0.01 0.01  8.78e-02
## G_std:scale(Age_Center_0)              0.00 0.00  2.06e-01
## Sex:scale(Age_Center_0)               -0.01 0.00  2.00e-01
## G_std:I(scale(Age_Center_0)^2)         0.00 0.00  6.45e-01
## Sex:I(scale(Age_Center_0)^2)          -0.01 0.00  1.39e-01
## G_std:Sex:scale(Age_Center_0)          0.00 0.00  7.29e-01
## G_std:Sex:I(scale(Age_Center_0)^2)     0.00 0.00  2.08e-01
```

## 4. Probability of having a phenotype as a function of the g-factor

```
## `geom_smooth()` using formula 'y ~ x'
```
