# Supplementary material for: High intelligence is not associated with a greater propensity for mental health disorders
Source: Eur Psychiatry. 2022 Nov 18;66(1):e3. doi: 10.1192/j.eurpsy.2022.2343 (PMC9879926; doi:10.1192/j.eurpsy.2022.2343)
Supplement: Supplementary file 1 [file S0924933822023434sup001.zip › S0924933822023434sup012.html]

Same Sex Sexual Behavior


# Same Sex Sexual Behavior

#### 2022-09-21

## 1. Regression with g-factor Group

```
## [1] "There are 239847 individuals without missing data in this analysis."
```

```
##                                                               Estimate   SE
## (Intercept)                                                      -3.28 0.02
## CA_GroupHigh_CA                                                   0.14 0.06
## CA_GroupLow_CA                                                    0.10 0.09
## Sex                                                              -0.51 0.03
## scale(Age_Center_0, scale = FALSE)                               -0.06 0.00
## I(scale((Age_Center_0), scale = FALSE)^2)                         0.00 0.00
## CA_GroupHigh_CA:Sex                                               0.36 0.12
## CA_GroupLow_CA:Sex                                               -0.57 0.18
## CA_GroupHigh_CA:scale(Age_Center_0, scale = FALSE)                0.01 0.01
## CA_GroupLow_CA:scale(Age_Center_0, scale = FALSE)                 0.00 0.01
## Sex:scale(Age_Center_0, scale = FALSE)                           -0.03 0.00
## CA_GroupHigh_CA:I(scale((Age_Center_0), scale = FALSE)^2)         0.00 0.00
## CA_GroupLow_CA:I(scale((Age_Center_0), scale = FALSE)^2)          0.00 0.00
## Sex:I(scale((Age_Center_0), scale = FALSE)^2)                     0.00 0.00
## CA_GroupHigh_CA:Sex:scale(Age_Center_0, scale = FALSE)            0.01 0.01
## CA_GroupLow_CA:Sex:scale(Age_Center_0, scale = FALSE)            -0.01 0.02
## CA_GroupHigh_CA:Sex:I(scale((Age_Center_0), scale = FALSE)^2)     0.00 0.00
## CA_GroupLow_CA:Sex:I(scale((Age_Center_0), scale = FALSE)^2)      0.00 0.00
##                                                                       p   OR
## (Intercept)                                                    0.00e+00 0.04
## CA_GroupHigh_CA                                                1.26e-02 1.15
## CA_GroupLow_CA                                                 2.49e-01 1.11
## Sex                                                            6.80e-55 0.60
## scale(Age_Center_0, scale = FALSE)                            1.43e-232 0.94
## I(scale((Age_Center_0), scale = FALSE)^2)                      2.16e-05 1.00
## CA_GroupHigh_CA:Sex                                            2.06e-03 1.43
## CA_GroupLow_CA:Sex                                             1.13e-03 0.57
## CA_GroupHigh_CA:scale(Age_Center_0, scale = FALSE)             2.50e-01 1.01
## CA_GroupLow_CA:scale(Age_Center_0, scale = FALSE)              6.71e-01 1.00
## Sex:scale(Age_Center_0, scale = FALSE)                         5.81e-16 0.97
## CA_GroupHigh_CA:I(scale((Age_Center_0), scale = FALSE)^2)      7.11e-01 1.00
## CA_GroupLow_CA:I(scale((Age_Center_0), scale = FALSE)^2)       8.44e-01 1.00
## Sex:I(scale((Age_Center_0), scale = FALSE)^2)                  9.24e-03 1.00
## CA_GroupHigh_CA:Sex:scale(Age_Center_0, scale = FALSE)         6.29e-01 1.01
## CA_GroupLow_CA:Sex:scale(Age_Center_0, scale = FALSE)          7.61e-01 0.99
## CA_GroupHigh_CA:Sex:I(scale((Age_Center_0), scale = FALSE)^2)  6.97e-01 1.00
## CA_GroupLow_CA:Sex:I(scale((Age_Center_0), scale = FALSE)^2)   5.56e-01 1.00
```

## 2. CA Group Regression Assumptions

### a) Influential values

There is no influential observations in our data.

```
## # A tibble: 3 × 13
##   .rownames Same_Sex_Behavior_0 CA_Group   Sex `scale(Age…`[,1] `I(scale((…`[,1]
##   <chr>                   <int> <fct>    <dbl>            <dbl>         <I<dbl>>
## 1 61797                       1 Low_CA     0.5             12.3             152.
## 2 71948                       1 Low_CA     0.5             12.2             148.
## 3 244815                      1 Low_CA     0.5             11.8             140.
## # … with 7 more variables: .fitted <dbl>, .resid <dbl>, .std.resid <dbl>,
## #   .hat <dbl>, .sigma <dbl>, .cooksd <dbl>, index <int>
```

```
## # A tibble: 86 × 13
##    .rownames Same_Sex_Behavior… CA_Group   Sex `scale(Age…`[,1] `I(scale((…`[,1]
##    <chr>                  <int> <fct>    <dbl>            <dbl>         <I<dbl>>
##  1 2035                       1 Average…   0.5            11.5             132. 
##  2 3763                       1 Low_CA     0.5            11.0             121. 
##  3 6446                       1 Average…   0.5            13.3             178. 
##  4 8911                       1 Average…   0.5            12.4             154. 
##  5 17930                      1 Average…   0.5            12.9             167. 
##  6 19671                      1 Average…   0.5            12.9             167. 
##  7 25090                      1 Average…   0.5            11.0             121. 
##  8 28074                      1 Low_CA     0.5             9.01             81.1
##  9 37634                      1 Average…   0.5            11.4             130. 
## 10 42431                      1 Average…   0.5            11.5             132. 
## # … with 76 more rows, and 7 more variables: .fitted <dbl>, .resid <dbl>,
## #   .std.resid <dbl>, .hat <dbl>, .sigma <dbl>, .cooksd <dbl>, index <int>
```

### b) Multicollinearity

As a rule of thumb, a VIF value that exceeds 5 or 10 indicates a
problematic amount of collinearity.

```
## there are higher-order terms (interactions) in this model
## consider setting terms = 'marginal' or 'high-order'; see ?vif
```

```
##                                                             GVIF Df
## CA_Group                                                4.887317  2
## Sex                                                     2.319507  1
## scale(Age_Center_0, scale = FALSE)                      1.884005  1
## I(scale((Age_Center_0), scale = FALSE)^2)               1.772423  1
## CA_Group:Sex                                            5.455021  2
## CA_Group:scale(Age_Center_0, scale = FALSE)             5.693867  2
## Sex:scale(Age_Center_0, scale = FALSE)                  2.162439  1
## CA_Group:I(scale((Age_Center_0), scale = FALSE)^2)     13.789572  2
## Sex:I(scale((Age_Center_0), scale = FALSE)^2)           3.563730  1
## CA_Group:Sex:scale(Age_Center_0, scale = FALSE)         5.780792  2
## CA_Group:Sex:I(scale((Age_Center_0), scale = FALSE)^2) 14.529879  2
##                                                        GVIF^(1/(2*Df))
## CA_Group                                                      1.486852
## Sex                                                           1.522993
## scale(Age_Center_0, scale = FALSE)                            1.372590
## I(scale((Age_Center_0), scale = FALSE)^2)                     1.331324
## CA_Group:Sex                                                  1.528267
## CA_Group:scale(Age_Center_0, scale = FALSE)                   1.544727
## Sex:scale(Age_Center_0, scale = FALSE)                        1.470523
## CA_Group:I(scale((Age_Center_0), scale = FALSE)^2)            1.927027
## Sex:I(scale((Age_Center_0), scale = FALSE)^2)                 1.887784
## CA_Group:Sex:scale(Age_Center_0, scale = FALSE)               1.550589
## CA_Group:Sex:I(scale((Age_Center_0), scale = FALSE)^2)        1.952385
```

## 3. Regression with g-factor

```
## [1] "There are 239847 individuals without missing data in this analysis."
```

```
##                                               Estimate   SE         p   OR
## (Intercept)                                      -3.27 0.02  0.00e+00 0.04
## G_std                                             0.03 0.01  7.50e-03 1.03
## Sex                                              -0.61 0.02 1.91e-141 0.54
## scale(Age_Center_0, scale = FALSE)               -0.06 0.00 6.10e-259 0.94
## I(scale(Age_Center_0, scale = FALSE)^2)           0.00 0.00  1.57e-04 1.00
## G_std:Sex                                         0.23 0.02  9.72e-30 1.26
## G_std:scale(Age_Center_0, scale = FALSE)          0.00 0.00  4.35e-03 1.00
## Sex:scale(Age_Center_0, scale = FALSE)           -0.02 0.00  2.26e-18 0.98
## G_std:I(scale(Age_Center_0, scale = FALSE)^2)     0.00 0.00  5.74e-01 1.00
## G_std:Sex:scale(Age_Center_0, scale = FALSE)      0.01 0.00  1.15e-03 1.01
```

## 4. Probability of having a phenotype as a function of the g-factor

### a) Without data points

```
## Using data Same_Sex_Behavior_DF_items_0 from global environment. This could
## cause incorrect results if Same_Sex_Behavior_DF_items_0 has been altered
## since the model was fit. You can manually provide the data to the "data ="
## argument.
```

### b) With data points

```
## Using data Same_Sex_Behavior_DF_items_0 from global environment. This could
## cause incorrect results if Same_Sex_Behavior_DF_items_0 has been altered
## since the model was fit. You can manually provide the data to the "data ="
## argument.
```
