# Supplementary material for: High intelligence is not associated with a greater propensity for mental health disorders
Source: Eur Psychiatry. 2022 Nov 18;66(1):e3. doi: 10.1192/j.eurpsy.2022.2343 (PMC9879926; doi:10.1192/j.eurpsy.2022.2343)
Supplement: Supplementary file 1 [file S0924933822023434sup001.zip › S0924933822023434sup013.html]

Insomnia


# Insomnia

#### 2022-09-21

## 1. CA Group Regressions

```
## [1] "There are 261518 individuals without missing data in this analysis."
```

```
##                                                                   Estimate   SE
## (Intercept)                                                           1.32 0.01
## CA_GroupHigh_CA                                                      -0.01 0.03
## CA_GroupLow_CA                                                        0.08 0.04
## Sex                                                                   0.81 0.01
## scale(max_age_insomnia, scale = FALSE)                                0.03 0.00
## I(scale((max_age_insomnia), scale = FALSE)^2)                         0.00 0.00
## CA_GroupHigh_CA:Sex                                                  -0.03 0.05
## CA_GroupLow_CA:Sex                                                   -0.11 0.07
## CA_GroupHigh_CA:scale(max_age_insomnia, scale = FALSE)                0.01 0.00
## CA_GroupLow_CA:scale(max_age_insomnia, scale = FALSE)                -0.01 0.00
## Sex:scale(max_age_insomnia, scale = FALSE)                            0.01 0.00
## CA_GroupHigh_CA:I(scale((max_age_insomnia), scale = FALSE)^2)         0.00 0.00
## CA_GroupLow_CA:I(scale((max_age_insomnia), scale = FALSE)^2)          0.00 0.00
## Sex:I(scale((max_age_insomnia), scale = FALSE)^2)                     0.00 0.00
## CA_GroupHigh_CA:Sex:scale(max_age_insomnia, scale = FALSE)            0.00 0.00
## CA_GroupLow_CA:Sex:scale(max_age_insomnia, scale = FALSE)             0.00 0.01
## CA_GroupHigh_CA:Sex:I(scale((max_age_insomnia), scale = FALSE)^2)     0.00 0.00
## CA_GroupLow_CA:Sex:I(scale((max_age_insomnia), scale = FALSE)^2)      0.00 0.00
##                                                                          p   OR
## (Intercept)                                                       0.00e+00 3.74
## CA_GroupHigh_CA                                                   7.91e-01 0.99
## CA_GroupLow_CA                                                    3.00e-02 1.08
## Sex                                                               0.00e+00 2.25
## scale(max_age_insomnia, scale = FALSE)                            0.00e+00 1.03
## I(scale((max_age_insomnia), scale = FALSE)^2)                     1.40e-07 1.00
## CA_GroupHigh_CA:Sex                                               6.10e-01 0.97
## CA_GroupLow_CA:Sex                                                1.23e-01 0.90
## CA_GroupHigh_CA:scale(max_age_insomnia, scale = FALSE)            1.62e-02 1.01
## CA_GroupLow_CA:scale(max_age_insomnia, scale = FALSE)             5.39e-03 0.99
## Sex:scale(max_age_insomnia, scale = FALSE)                        3.72e-24 1.01
## CA_GroupHigh_CA:I(scale((max_age_insomnia), scale = FALSE)^2)     6.56e-01 1.00
## CA_GroupLow_CA:I(scale((max_age_insomnia), scale = FALSE)^2)      7.01e-01 1.00
## Sex:I(scale((max_age_insomnia), scale = FALSE)^2)                 2.76e-44 1.00
## CA_GroupHigh_CA:Sex:scale(max_age_insomnia, scale = FALSE)        6.63e-01 1.00
## CA_GroupLow_CA:Sex:scale(max_age_insomnia, scale = FALSE)         5.19e-01 1.00
## CA_GroupHigh_CA:Sex:I(scale((max_age_insomnia), scale = FALSE)^2) 1.94e-01 1.00
## CA_GroupLow_CA:Sex:I(scale((max_age_insomnia), scale = FALSE)^2)  5.12e-01 1.00
```

## 2. Regression with g-factor Group Assumptions

### a) Influential values

There is no influential observations in our data.

```
## # A tibble: 3 × 12
##   Insomnia CA_Group   Sex `scale(max_age_i…`[,1] `I(scale((…`[,1] .fitted .resid
##      <int> <fct>    <dbl>                  <dbl>         <I<dbl>>   <dbl>  <dbl>
## 1        0 Low_CA     0.5                   22.1             490.    2.06  -2.09
## 2        0 Low_CA     0.5                   20.1             405.    2.07  -2.09
## 3        0 Low_CA     0.5                   21.2             450.    2.07  -2.09
## # … with 5 more variables: .std.resid <dbl>, .hat <dbl>, .sigma <dbl>,
## #   .cooksd <dbl>, index <int>
```

```
## # A tibble: 0 × 12
## # … with 12 variables: Insomnia <int>, CA_Group <fct>, Sex <dbl>,
## #   scale(max_age_insomnia, scale = FALSE) <dbl[,1]>,
## #   I(scale((max_age_insomnia), scale = FALSE)^2) <I<dbl[,1]>[,1]>,
## #   .fitted <dbl>, .resid <dbl>, .std.resid <dbl>, .hat <dbl>, .sigma <dbl>,
## #   .cooksd <dbl>, index <int>
```

### b) Multicollinearity

As a rule of thumb, a VIF value that exceeds 5 or 10 indicates a
problematic amount of collinearity.

```
## there are higher-order terms (interactions) in this model
## consider setting terms = 'marginal' or 'high-order'; see ?vif
```

```
##                                                                GVIF Df
## CA_Group                                                   3.593480  2
## Sex                                                        2.023036  1
## scale(max_age_insomnia, scale = FALSE)                     1.346822  1
## I(scale((max_age_insomnia), scale = FALSE)^2)              1.327494  1
## CA_Group:Sex                                               3.965183  2
## CA_Group:scale(max_age_insomnia, scale = FALSE)            1.720304  2
## Sex:scale(max_age_insomnia, scale = FALSE)                 1.363791  1
## CA_Group:I(scale((max_age_insomnia), scale = FALSE)^2)     4.844164  2
## Sex:I(scale((max_age_insomnia), scale = FALSE)^2)          2.371873  1
## CA_Group:Sex:scale(max_age_insomnia, scale = FALSE)        1.723317  2
## CA_Group:Sex:I(scale((max_age_insomnia), scale = FALSE)^2) 5.053809  2
##                                                            GVIF^(1/(2*Df))
## CA_Group                                                          1.376825
## Sex                                                               1.422335
## scale(max_age_insomnia, scale = FALSE)                            1.160526
## I(scale((max_age_insomnia), scale = FALSE)^2)                     1.152169
## CA_Group:Sex                                                      1.411126
## CA_Group:scale(max_age_insomnia, scale = FALSE)                   1.145253
## Sex:scale(max_age_insomnia, scale = FALSE)                        1.167815
## CA_Group:I(scale((max_age_insomnia), scale = FALSE)^2)            1.483559
## Sex:I(scale((max_age_insomnia), scale = FALSE)^2)                 1.540089
## CA_Group:Sex:scale(max_age_insomnia, scale = FALSE)               1.145754
## CA_Group:Sex:I(scale((max_age_insomnia), scale = FALSE)^2)        1.499356
```

## 3. Regression with g-factor

```
## [1] "There are 261518 individuals without missing data in this analysis."
```

```
##                                                   Estimate   SE        p   OR
## (Intercept)                                           1.32 0.01 0.00e+00 3.74
## G_std                                                -0.01 0.01 1.22e-01 0.99
## Sex                                                   0.67 0.01 0.00e+00 1.95
## scale(max_age_insomnia, scale = FALSE)                0.04 0.00 0.00e+00 1.04
## I(scale(max_age_insomnia, scale = FALSE)^2)           0.00 0.00 3.17e-03 1.00
## G_std:Sex                                             0.00 0.01 8.39e-01 1.00
## G_std:scale(max_age_insomnia, scale = FALSE)          0.00 0.00 7.43e-14 1.00
## Sex:scale(max_age_insomnia, scale = FALSE)            0.02 0.00 1.63e-71 1.02
## G_std:I(scale(max_age_insomnia, scale = FALSE)^2)     0.00 0.00 3.81e-01 1.00
## G_std:Sex:scale(max_age_insomnia, scale = FALSE)      0.00 0.00 6.39e-01 1.00
```

## 4. Probability of having a phenotype as a function of the g-factor

### a) Without data points

```
## Using data Sleep_DF_no_NA from global environment. This could cause
## incorrect results if Sleep_DF_no_NA has been altered since the model was
## fit. You can manually provide the data to the "data =" argument.
```

### b) With data points

```
## Using data Sleep_DF_no_NA from global environment. This could cause
## incorrect results if Sleep_DF_no_NA has been altered since the model was
## fit. You can manually provide the data to the "data =" argument.
```
