# Supplementary material for: High intelligence is not associated with a greater propensity for mental health disorders
Source: Eur Psychiatry. 2022 Nov 18;66(1):e3. doi: 10.1192/j.eurpsy.2022.2343 (PMC9879926; doi:10.1192/j.eurpsy.2022.2343)
Supplement: Supplementary file 1 [file S0924933822023434sup001.zip › S0924933822023434sup014.html]

Hay Fever Rhinitis


# Hay Fever Rhinitis

#### 2022-09-21

## 1. CA Group Regressions

```
## [1] "There are 102837 individuals without missing data in this analysis."
```

```
##                                                              Estimate   SE
## (Intercept)                                                     -1.11 0.01
## CA_GroupHigh_CA                                                  0.12 0.03
## CA_GroupLow_CA                                                  -0.21 0.07
## Sex                                                              0.08 0.02
## scale(max_age_HFR, scale = FALSE)                               -0.03 0.00
## I(scale((max_age_HFR), scale = FALSE)^2)                         0.00 0.00
## CA_GroupHigh_CA:Sex                                              0.06 0.07
## CA_GroupLow_CA:Sex                                               0.00 0.15
## CA_GroupHigh_CA:scale(max_age_HFR, scale = FALSE)                0.00 0.00
## CA_GroupLow_CA:scale(max_age_HFR, scale = FALSE)                 0.01 0.01
## Sex:scale(max_age_HFR, scale = FALSE)                            0.00 0.00
## CA_GroupHigh_CA:I(scale((max_age_HFR), scale = FALSE)^2)         0.00 0.00
## CA_GroupLow_CA:I(scale((max_age_HFR), scale = FALSE)^2)          0.00 0.00
## Sex:I(scale((max_age_HFR), scale = FALSE)^2)                     0.00 0.00
## CA_GroupHigh_CA:Sex:scale(max_age_HFR, scale = FALSE)           -0.01 0.01
## CA_GroupLow_CA:Sex:scale(max_age_HFR, scale = FALSE)             0.02 0.01
## CA_GroupHigh_CA:Sex:I(scale((max_age_HFR), scale = FALSE)^2)     0.00 0.00
## CA_GroupLow_CA:Sex:I(scale((max_age_HFR), scale = FALSE)^2)      0.00 0.00
##                                                                      p   OR
## (Intercept)                                                   0.00e+00 0.33
## CA_GroupHigh_CA                                               2.90e-04 1.13
## CA_GroupLow_CA                                                5.23e-03 0.81
## Sex                                                           3.40e-04 1.08
## scale(max_age_HFR, scale = FALSE)                            6.84e-134 0.97
## I(scale((max_age_HFR), scale = FALSE)^2)                      1.65e-03 1.00
## CA_GroupHigh_CA:Sex                                           3.91e-01 1.06
## CA_GroupLow_CA:Sex                                            9.90e-01 1.00
## CA_GroupHigh_CA:scale(max_age_HFR, scale = FALSE)             1.49e-01 1.00
## CA_GroupLow_CA:scale(max_age_HFR, scale = FALSE)              4.44e-01 1.01
## Sex:scale(max_age_HFR, scale = FALSE)                         2.88e-01 1.00
## CA_GroupHigh_CA:I(scale((max_age_HFR), scale = FALSE)^2)      6.28e-01 1.00
## CA_GroupLow_CA:I(scale((max_age_HFR), scale = FALSE)^2)       2.39e-01 1.00
## Sex:I(scale((max_age_HFR), scale = FALSE)^2)                  3.48e-01 1.00
## CA_GroupHigh_CA:Sex:scale(max_age_HFR, scale = FALSE)         2.27e-01 0.99
## CA_GroupLow_CA:Sex:scale(max_age_HFR, scale = FALSE)          2.91e-01 1.02
## CA_GroupHigh_CA:Sex:I(scale((max_age_HFR), scale = FALSE)^2)  8.01e-02 1.00
## CA_GroupLow_CA:Sex:I(scale((max_age_HFR), scale = FALSE)^2)   9.18e-01 1.00
```

## 2. Regression with g-factor Group Assumptions

### a) Influential values

There is no influential observations in our data.

```
## # A tibble: 3 × 12
##   Phenotype_HFR_0_3 CA_Group   Sex `scale(max_age…`[,1] `I(scale((…`[,1] .fitted
##               <dbl> <fct>    <dbl>                <dbl>         <I<dbl>>   <dbl>
## 1                 1 Low_CA    -0.5                -17.7             314.  -0.622
## 2                 1 Low_CA    -0.5                 14.0             197.  -1.66 
## 3                 1 Low_CA    -0.5                 16.6             276.  -1.69 
## # … with 6 more variables: .resid <dbl>, .std.resid <dbl>, .hat <dbl>,
## #   .sigma <dbl>, .cooksd <dbl>, index <int>
```

```
## # A tibble: 0 × 12
## # … with 12 variables: Phenotype_HFR_0_3 <dbl>, CA_Group <fct>, Sex <dbl>,
## #   scale(max_age_HFR, scale = FALSE) <dbl[,1]>,
## #   I(scale((max_age_HFR), scale = FALSE)^2) <I<dbl[,1]>[,1]>, .fitted <dbl>,
## #   .resid <dbl>, .std.resid <dbl>, .hat <dbl>, .sigma <dbl>, .cooksd <dbl>,
## #   index <int>
```

### b) Multicollinearity

As a rule of thumb, a VIF value that exceeds 5 or 10 indicates a
problematic amount of collinearity.

```
## there are higher-order terms (interactions) in this model
## consider setting terms = 'marginal' or 'high-order'; see ?vif
```

```
##                                                           GVIF Df
## CA_Group                                              3.775563  2
## Sex                                                   2.182827  1
## scale(max_age_HFR, scale = FALSE)                     1.362722  1
## I(scale((max_age_HFR), scale = FALSE)^2)              1.375691  1
## CA_Group:Sex                                          4.299687  2
## CA_Group:scale(max_age_HFR, scale = FALSE)            1.611461  2
## Sex:scale(max_age_HFR, scale = FALSE)                 1.365159  1
## CA_Group:I(scale((max_age_HFR), scale = FALSE)^2)     4.905857  2
## Sex:I(scale((max_age_HFR), scale = FALSE)^2)          2.474886  1
## CA_Group:Sex:scale(max_age_HFR, scale = FALSE)        1.612369  2
## CA_Group:Sex:I(scale((max_age_HFR), scale = FALSE)^2) 5.185142  2
##                                                       GVIF^(1/(2*Df))
## CA_Group                                                     1.393944
## Sex                                                          1.477439
## scale(max_age_HFR, scale = FALSE)                            1.167357
## I(scale((max_age_HFR), scale = FALSE)^2)                     1.172898
## CA_Group:Sex                                                 1.439989
## CA_Group:scale(max_age_HFR, scale = FALSE)                   1.126691
## Sex:scale(max_age_HFR, scale = FALSE)                        1.168400
## CA_Group:I(scale((max_age_HFR), scale = FALSE)^2)            1.488260
## Sex:I(scale((max_age_HFR), scale = FALSE)^2)                 1.573177
## CA_Group:Sex:scale(max_age_HFR, scale = FALSE)               1.126850
## CA_Group:Sex:I(scale((max_age_HFR), scale = FALSE)^2)        1.509003
```

## 3. Regression with g-factor

```
## [1] "There are 102837 individuals without missing data in this analysis."
```

```
##                                              Estimate   SE         p   OR
## (Intercept)                                     -1.14 0.01  0.00e+00 0.32
## G_std                                            0.06 0.01  5.87e-15 1.06
## Sex                                              0.07 0.02  3.32e-06 1.07
## scale(max_age_HFR, scale = FALSE)               -0.03 0.00 2.07e-126 0.97
## I(scale(max_age_HFR, scale = FALSE)^2)           0.00 0.00  1.21e-02 1.00
## G_std:Sex                                       -0.01 0.01  2.74e-01 0.99
## G_std:scale(max_age_HFR, scale = FALSE)          0.00 0.00  5.88e-01 1.00
## Sex:scale(max_age_HFR, scale = FALSE)            0.00 0.00  5.82e-02 1.00
## G_std:I(scale(max_age_HFR, scale = FALSE)^2)     0.00 0.00  5.09e-01 1.00
## G_std:Sex:scale(max_age_HFR, scale = FALSE)      0.00 0.00  2.60e-01 1.00
```

## 4. Probability of having a phenotype as a function of the g-factor

### a) Without data points

```
## Using data Phenotypes_Allergies_HFR_0_1_2_3_no_na from global environment.
## This could cause incorrect results if
## Phenotypes_Allergies_HFR_0_1_2_3_no_na has been altered since the model was
## fit. You can manually provide the data to the "data =" argument.
```

### b) With data points

```
## Using data Phenotypes_Allergies_HFR_0_1_2_3_no_na from global environment.
## This could cause incorrect results if
## Phenotypes_Allergies_HFR_0_1_2_3_no_na has been altered since the model was
## fit. You can manually provide the data to the "data =" argument.
```
