# Supplementary material for: High intelligence is not associated with a greater propensity for mental health disorders
Source: Eur Psychiatry. 2022 Nov 18;66(1):e3. doi: 10.1192/j.eurpsy.2022.2343 (PMC9879926; doi:10.1192/j.eurpsy.2022.2343)
Supplement: Supplementary file 1 [file S0924933822023434sup001.zip › S0924933822023434sup015.html]

Anxiety


# Anxiety

#### 2022-09-21

## 1. Regression with g-factor Group

```
## [1] "There are 81892 individuals without missing data in this analysis."
```

```
##                                                    Estimate   SE         p   OR
## (Intercept)                                           -1.71 0.01  0.00e+00 0.18
## CA_GroupHigh_CA                                       -0.37 0.05  7.00e-13 0.69
## CA_GroupLow_CA                                         0.27 0.09  1.36e-03 1.31
## Sex                                                    0.59 0.02 1.30e-159 1.80
## scale(max_age, scale = FALSE)                         -0.03 0.00  9.34e-84 0.97
## I(scale(max_age, scale = FALSE)^2)                     0.00 0.00  4.89e-11 1.00
## CA_GroupHigh_CA:Sex                                   -0.11 0.08  1.66e-01 0.90
## CA_GroupLow_CA:Sex                                     0.02 0.13  9.00e-01 1.02
## CA_GroupHigh_CA:scale(max_age, scale = FALSE)          0.00 0.01  4.20e-01 1.00
## CA_GroupLow_CA:scale(max_age, scale = FALSE)          -0.02 0.01  8.58e-02 0.98
## Sex:scale(max_age, scale = FALSE)                      0.00 0.00  6.19e-01 1.00
## CA_GroupHigh_CA:I(scale(max_age, scale = FALSE)^2)     0.00 0.00  3.47e-01 1.00
## CA_GroupLow_CA:I(scale(max_age, scale = FALSE)^2)      0.00 0.00  7.64e-01 1.00
## CA_GroupHigh_CA:Sex:scale(max_age, scale = FALSE)     -0.01 0.01  3.01e-01 0.99
## CA_GroupLow_CA:Sex:scale(max_age, scale = FALSE)       0.01 0.02  6.27e-01 1.01
```

## 2. CA Group Regression Assumptions

### a) Influential values

There are no influential observations in our data.

```
## # A tibble: 3 × 12
##   Anxiety_General CA_Group   Sex `scale(max_age, …`[,1] `I(scale(m…`[,1] .fitted
##             <dbl> <fct>    <dbl>                  <dbl>         <I<dbl>>   <dbl>
## 1               1 Low_CA    -0.5                   12.5             155.   -2.48
## 2               1 Low_CA    -0.5                   13.3             177.   -2.54
## 3               1 Low_CA    -0.5                   14.0             197.   -2.60
## # … with 6 more variables: .resid <dbl>, .std.resid <dbl>, .hat <dbl>,
## #   .sigma <dbl>, .cooksd <dbl>, index <int>
```

```
## # A tibble: 0 × 12
## # … with 12 variables: Anxiety_General <dbl>, CA_Group <fct>, Sex <dbl>,
## #   scale(max_age, scale = FALSE) <dbl[,1]>,
## #   I(scale(max_age, scale = FALSE)^2) <I<dbl[,1]>[,1]>, .fitted <dbl>,
## #   .resid <dbl>, .std.resid <dbl>, .hat <dbl>, .sigma <dbl>, .cooksd <dbl>,
## #   index <int>
```

```
##                                                         Estimate           SE
## (Intercept)                                        -1.7096532973 0.0145874176
## CA_GroupHigh_CA                                    -0.3715564109 0.0517524601
## CA_GroupLow_CA                                      0.2726478694 0.0851273947
## Sex                                                 0.5908864083 0.0219502041
## scale(max_age, scale = FALSE)                      -0.0305604438 0.0015760772
## I(scale(max_age, scale = FALSE)^2)                 -0.0011485495 0.0001747033
## CA_GroupHigh_CA:Sex                                -0.1087059636 0.0784496936
## CA_GroupLow_CA:Sex                                  0.0160374850 0.1280505566
## CA_GroupHigh_CA:scale(max_age, scale = FALSE)       0.0045155111 0.0056002289
## CA_GroupLow_CA:scale(max_age, scale = FALSE)       -0.0152083516 0.0088531749
## Sex:scale(max_age, scale = FALSE)                  -0.0014179091 0.0028517743
## CA_GroupHigh_CA:I(scale(max_age, scale = FALSE)^2)  0.0005715768 0.0006075441
## CA_GroupLow_CA:I(scale(max_age, scale = FALSE)^2)   0.0002953636 0.0009834927
## CA_GroupHigh_CA:Sex:scale(max_age, scale = FALSE)  -0.0103007387 0.0099513358
## CA_GroupLow_CA:Sex:scale(max_age, scale = FALSE)    0.0078194359 0.0160771939
##                                                             t/z             p
## (Intercept)                                        -117.2005455  0.000000e+00
## CA_GroupHigh_CA                                      -7.1794927  6.997055e-13
## CA_GroupLow_CA                                        3.2028217  1.360882e-03
## Sex                                                  26.9194039 1.301950e-159
## scale(max_age, scale = FALSE)                       -19.3901950  9.338226e-84
## I(scale(max_age, scale = FALSE)^2)                   -6.5742881  4.888649e-11
## CA_GroupHigh_CA:Sex                                  -1.3856774  1.658454e-01
## CA_GroupLow_CA:Sex                                    0.1252434  9.003309e-01
## CA_GroupHigh_CA:scale(max_age, scale = FALSE)         0.8063083  4.200651e-01
## CA_GroupLow_CA:scale(max_age, scale = FALSE)         -1.7178415  8.582552e-02
## Sex:scale(max_age, scale = FALSE)                    -0.4972024  6.190463e-01
## CA_GroupHigh_CA:I(scale(max_age, scale = FALSE)^2)    0.9407990  3.468079e-01
## CA_GroupLow_CA:I(scale(max_age, scale = FALSE)^2)     0.3003211  7.639323e-01
## CA_GroupHigh_CA:Sex:scale(max_age, scale = FALSE)    -1.0351112  3.006170e-01
## CA_GroupLow_CA:Sex:scale(max_age, scale = FALSE)      0.4863682  6.267061e-01
##                                                                  Model
## (Intercept)                                        Anxiety_General 0_3
## CA_GroupHigh_CA                                    Anxiety_General 0_3
## CA_GroupLow_CA                                     Anxiety_General 0_3
## Sex                                                Anxiety_General 0_3
## scale(max_age, scale = FALSE)                      Anxiety_General 0_3
## I(scale(max_age, scale = FALSE)^2)                 Anxiety_General 0_3
## CA_GroupHigh_CA:Sex                                Anxiety_General 0_3
## CA_GroupLow_CA:Sex                                 Anxiety_General 0_3
## CA_GroupHigh_CA:scale(max_age, scale = FALSE)      Anxiety_General 0_3
## CA_GroupLow_CA:scale(max_age, scale = FALSE)       Anxiety_General 0_3
## Sex:scale(max_age, scale = FALSE)                  Anxiety_General 0_3
## CA_GroupHigh_CA:I(scale(max_age, scale = FALSE)^2) Anxiety_General 0_3
## CA_GroupLow_CA:I(scale(max_age, scale = FALSE)^2)  Anxiety_General 0_3
## CA_GroupHigh_CA:Sex:scale(max_age, scale = FALSE)  Anxiety_General 0_3
## CA_GroupLow_CA:Sex:scale(max_age, scale = FALSE)   Anxiety_General 0_3
```

### b) Multicollinearity

As a rule of thumb, a VIF value that exceeds 5 or 10 indicates a
problematic amount of collinearity.

```
## there are higher-order terms (interactions) in this model
## consider setting terms = 'marginal' or 'high-order'; see ?vif
```

```
##                                                 GVIF Df GVIF^(1/(2*Df))
## CA_Group                                    3.756112  2        1.392146
## Sex                                         1.145778  1        1.070410
## scale(max_age, scale = FALSE)               1.484923  1        1.218574
## I(scale(max_age, scale = FALSE)^2)          1.423550  1        1.193126
## CA_Group:Sex                                1.353232  2        1.078557
## CA_Group:scale(max_age, scale = FALSE)      2.022530  2        1.192542
## Sex:scale(max_age, scale = FALSE)           1.231281  1        1.109631
## CA_Group:I(scale(max_age, scale = FALSE)^2) 5.538318  2        1.534068
## CA_Group:Sex:scale(max_age, scale = FALSE)  1.318745  2        1.071618
```

## 3. Regression with g-factor

```
## [1] "There are 81892 individuals without missing data in this analysis."
```

```
##                                          Estimate   SE         p   OR
## (Intercept)                                 -1.68 0.01  0.00e+00 0.19
## G_std                                       -0.14 0.01  8.40e-35 0.87
## Sex                                          0.57 0.02 3.98e-157 1.77
## scale(max_age, scale = FALSE)               -0.03 0.00  1.87e-96 0.97
## I(scale(max_age, scale = FALSE)^2)           0.00 0.00  1.32e-11 1.00
## G_std:Sex                                   -0.01 0.02  5.42e-01 0.99
## G_std:scale(max_age, scale = FALSE)          0.00 0.00  3.32e-02 1.00
## Sex:scale(max_age, scale = FALSE)            0.00 0.00  6.23e-01 1.00
## G_std:I(scale(max_age, scale = FALSE)^2)     0.00 0.00  5.97e-01 1.00
## G_std:Sex:scale(max_age, scale = FALSE)      0.00 0.00  4.22e-01 1.00
```

## 4. Probability of having a phenotype as a function of the g-factor

### a) Without data points

```
## Using data Anxiety_DF_items_0_3 from global environment. This could cause
## incorrect results if Anxiety_DF_items_0_3 has been altered since the model
## was fit. You can manually provide the data to the "data =" argument.
```

### b) With data points

```
## Using data Anxiety_DF_items_0_3 from global environment. This could cause
## incorrect results if Anxiety_DF_items_0_3 has been altered since the model
## was fit. You can manually provide the data to the "data =" argument.
```
