# Supplementary material for: High intelligence is not associated with a greater propensity for mental health disorders
Source: Eur Psychiatry. 2022 Nov 18;66(1):e3. doi: 10.1192/j.eurpsy.2022.2343 (PMC9879926; doi:10.1192/j.eurpsy.2022.2343)
Supplement: Supplementary file 1 [file S0924933822023434sup001.zip › S0924933822023434sup016.html]

Other Allergies


# Other Allergies

#### 2022-09-21

## 1. CA Group Regressions

```
age_sex_model_binary <- glm(Phenotype_Other_0_3 ~ CA_Group*Sex + CA_Group*scale(max_age_center_other, scale = FALSE)+
                              CA_Group*I(scale((max_age_center_other), scale = FALSE)^2), data = Phenotypes_Allergies_Other_0_3_no_na, 
                            family = binomial)
Summary_age_sex_model_binary_Phenotype_HFR <- as.data.frame(summary(age_sex_model_binary)$coefficients)
Summary_age_sex_model_binary_Phenotype_HFR$Model <- "Other"
names(Summary_age_sex_model_binary_Phenotype_HFR) <- c("Estimate", "SE", "t/z", "p", "Model")
Summary_age_sex_model_binary_Phenotype_HFR
```

```
##                                                                        Estimate
## (Intercept)                                                       -3.7921287677
## CA_GroupHigh_CA                                                    0.2823835967
## CA_GroupLow_CA                                                    -0.2365179009
## Sex                                                                0.5307244756
## scale(max_age_center_other, scale = FALSE)                         0.0248600207
## I(scale((max_age_center_other), scale = FALSE)^2)                  0.0009626929
## CA_GroupHigh_CA:Sex                                                0.0989722730
## CA_GroupLow_CA:Sex                                                 0.0785612398
## CA_GroupHigh_CA:scale(max_age_center_other, scale = FALSE)         0.0063111867
## CA_GroupLow_CA:scale(max_age_center_other, scale = FALSE)          0.0110834204
## CA_GroupHigh_CA:I(scale((max_age_center_other), scale = FALSE)^2) -0.0005033125
## CA_GroupLow_CA:I(scale((max_age_center_other), scale = FALSE)^2)   0.0001845002
##                                                                             SE
## (Intercept)                                                       0.0183428802
## CA_GroupHigh_CA                                                   0.0627160293
## CA_GroupLow_CA                                                    0.1058759816
## Sex                                                               0.0278057029
## scale(max_age_center_other, scale = FALSE)                        0.0015320477
## I(scale((max_age_center_other), scale = FALSE)^2)                 0.0001521481
## CA_GroupHigh_CA:Sex                                               0.0965827569
## CA_GroupLow_CA:Sex                                                0.1591448061
## CA_GroupHigh_CA:scale(max_age_center_other, scale = FALSE)        0.0055819034
## CA_GroupLow_CA:scale(max_age_center_other, scale = FALSE)         0.0084087821
## CA_GroupHigh_CA:I(scale((max_age_center_other), scale = FALSE)^2) 0.0005106482
## CA_GroupLow_CA:I(scale((max_age_center_other), scale = FALSE)^2)  0.0008233581
##                                                                            t/z
## (Intercept)                                                       -206.7357322
## CA_GroupHigh_CA                                                      4.5025745
## CA_GroupLow_CA                                                      -2.2339146
## Sex                                                                 19.0868930
## scale(max_age_center_other, scale = FALSE)                          16.2266627
## I(scale((max_age_center_other), scale = FALSE)^2)                    6.3273406
## CA_GroupHigh_CA:Sex                                                  1.0247406
## CA_GroupLow_CA:Sex                                                   0.4936463
## CA_GroupHigh_CA:scale(max_age_center_other, scale = FALSE)           1.1306514
## CA_GroupLow_CA:scale(max_age_center_other, scale = FALSE)            1.3180768
## CA_GroupHigh_CA:I(scale((max_age_center_other), scale = FALSE)^2)   -0.9856344
## CA_GroupLow_CA:I(scale((max_age_center_other), scale = FALSE)^2)     0.2240825
##                                                                              p
## (Intercept)                                                       0.000000e+00
## CA_GroupHigh_CA                                                   6.713520e-06
## CA_GroupLow_CA                                                    2.548869e-02
## Sex                                                               3.245051e-81
## scale(max_age_center_other, scale = FALSE)                        3.267484e-59
## I(scale((max_age_center_other), scale = FALSE)^2)                 2.494224e-10
## CA_GroupHigh_CA:Sex                                               3.054856e-01
## CA_GroupLow_CA:Sex                                                6.215560e-01
## CA_GroupHigh_CA:scale(max_age_center_other, scale = FALSE)        2.582019e-01
## CA_GroupLow_CA:scale(max_age_center_other, scale = FALSE)         1.874779e-01
## CA_GroupHigh_CA:I(scale((max_age_center_other), scale = FALSE)^2) 3.243125e-01
## CA_GroupLow_CA:I(scale((max_age_center_other), scale = FALSE)^2)  8.226931e-01
##                                                                   Model
## (Intercept)                                                       Other
## CA_GroupHigh_CA                                                   Other
## CA_GroupLow_CA                                                    Other
## Sex                                                               Other
## scale(max_age_center_other, scale = FALSE)                        Other
## I(scale((max_age_center_other), scale = FALSE)^2)                 Other
## CA_GroupHigh_CA:Sex                                               Other
## CA_GroupLow_CA:Sex                                                Other
## CA_GroupHigh_CA:scale(max_age_center_other, scale = FALSE)        Other
## CA_GroupLow_CA:scale(max_age_center_other, scale = FALSE)         Other
## CA_GroupHigh_CA:I(scale((max_age_center_other), scale = FALSE)^2) Other
## CA_GroupLow_CA:I(scale((max_age_center_other), scale = FALSE)^2)  Other
```

```
Summary_ChildA <- Summary_age_sex_model_binary_Phenotype_HFR
```

```
## [1] "There are 261395 individuals without missing data in this analysis."
```

```
##                                                                   Estimate   SE
## (Intercept)                                                          -3.79 0.02
## CA_GroupHigh_CA                                                       0.28 0.06
## CA_GroupLow_CA                                                       -0.24 0.11
## Sex                                                                   0.53 0.03
## scale(max_age_center_other, scale = FALSE)                            0.02 0.00
## I(scale((max_age_center_other), scale = FALSE)^2)                     0.00 0.00
## CA_GroupHigh_CA:Sex                                                   0.10 0.10
## CA_GroupLow_CA:Sex                                                    0.08 0.16
## CA_GroupHigh_CA:scale(max_age_center_other, scale = FALSE)            0.01 0.01
## CA_GroupLow_CA:scale(max_age_center_other, scale = FALSE)             0.01 0.01
## CA_GroupHigh_CA:I(scale((max_age_center_other), scale = FALSE)^2)     0.00 0.00
## CA_GroupLow_CA:I(scale((max_age_center_other), scale = FALSE)^2)      0.00 0.00
##                                                                          p   OR
## (Intercept)                                                       0.00e+00 0.02
## CA_GroupHigh_CA                                                   6.71e-06 1.32
## CA_GroupLow_CA                                                    2.55e-02 0.79
## Sex                                                               3.25e-81 1.70
## scale(max_age_center_other, scale = FALSE)                        3.27e-59 1.02
## I(scale((max_age_center_other), scale = FALSE)^2)                 2.49e-10 1.00
## CA_GroupHigh_CA:Sex                                               3.05e-01 1.11
## CA_GroupLow_CA:Sex                                                6.22e-01 1.08
## CA_GroupHigh_CA:scale(max_age_center_other, scale = FALSE)        2.58e-01 1.01
## CA_GroupLow_CA:scale(max_age_center_other, scale = FALSE)         1.87e-01 1.01
## CA_GroupHigh_CA:I(scale((max_age_center_other), scale = FALSE)^2) 3.24e-01 1.00
## CA_GroupLow_CA:I(scale((max_age_center_other), scale = FALSE)^2)  8.23e-01 1.00
```

## 2. Regression with g-factor Group Assumptions

### a) Influential values

There is no influential observations in our data.

```
## # A tibble: 3 × 12
##   Phenotype_Other_0_3 CA_Group   Sex `scale(max_a…`[,1] `I(scale((…`[,1] .fitted
##                 <dbl> <fct>    <dbl>              <dbl>         <I<dbl>>   <dbl>
## 1                   1 Low_CA     0.5               19.4             377.   -2.59
## 2                   1 Low_CA     0.5               19.8             393.   -2.56
## 3                   1 Low_CA    -0.5               19.5             380.   -3.20
## # … with 6 more variables: .resid <dbl>, .std.resid <dbl>, .hat <dbl>,
## #   .sigma <dbl>, .cooksd <dbl>, index <int>
```

```
## # A tibble: 17 × 12
##    Phenotype_Other_0_3 CA_Group   Sex `scale(max_…`[,1] `I(scale((…`[,1] .fitted
##                  <dbl> <fct>    <dbl>             <dbl>         <I<dbl>>   <dbl>
##  1                   1 Low_CA    -0.5             -5.67             32.1   -4.50
##  2                   1 Low_CA    -0.5             -6.42             41.2   -4.52
##  3                   1 Low_CA    -0.5            -13.8             191.    -4.61
##  4                   1 Low_CA    -0.5             -6.58             43.3   -4.52
##  5                   1 Low_CA    -0.5            -12.2             150.    -4.60
##  6                   1 Low_CA    -0.5            -10.8             117.    -4.59
##  7                   1 Low_CA    -0.5             -8.17             66.7   -4.55
##  8                   1 Low_CA    -0.5            -10.6             112.    -4.59
##  9                   1 Low_CA    -0.5            -15.6             243.    -4.61
## 10                   1 Low_CA    -0.5            -12.7             163.    -4.61
## 11                   1 Low_CA    -0.5            -10.2             105.    -4.58
## 12                   1 Low_CA    -0.5            -15.7             245.    -4.61
## 13                   1 Low_CA    -0.5             -9.00             81.0   -4.56
## 14                   1 Low_CA    -0.5             -9.08             82.5   -4.57
## 15                   1 Low_CA    -0.5            -14.3             205.    -4.61
## 16                   1 Low_CA    -0.5            -15.3             235.    -4.61
## 17                   1 Low_CA    -0.5            -15.6             243.    -4.61
## # … with 6 more variables: .resid <dbl>, .std.resid <dbl>, .hat <dbl>,
## #   .sigma <dbl>, .cooksd <dbl>, index <int>
```

### b) Multicollinearity

As a rule of thumb, a VIF value that exceeds 5 or 10 indicates a
problematic amount of collinearity.

```
## there are higher-order terms (interactions) in this model
## consider setting terms = 'marginal' or 'high-order'; see ?vif
```

```
##                                                                GVIF Df
## CA_Group                                                   3.668880  2
## Sex                                                        1.133104  1
## scale(max_age_center_other, scale = FALSE)                 1.124147  1
## I(scale((max_age_center_other), scale = FALSE)^2)          1.138919  1
## CA_Group:Sex                                               1.362645  2
## CA_Group:scale(max_age_center_other, scale = FALSE)        1.300912  2
## CA_Group:I(scale((max_age_center_other), scale = FALSE)^2) 3.486354  2
##                                                            GVIF^(1/(2*Df))
## CA_Group                                                          1.383991
## Sex                                                               1.064474
## scale(max_age_center_other, scale = FALSE)                        1.060258
## I(scale((max_age_center_other), scale = FALSE)^2)                 1.067202
## CA_Group:Sex                                                      1.080428
## CA_Group:scale(max_age_center_other, scale = FALSE)               1.067977
## CA_Group:I(scale((max_age_center_other), scale = FALSE)^2)        1.366447
```

## 3. Regression with g-factor

```
## [1] "There are 261395 individuals without missing data in this analysis."
```

```
##                                                       Estimate   SE        p
## (Intercept)                                              -3.80 0.02 0.00e+00
## G_std                                                     0.09 0.01 1.56e-09
## Sex                                                       0.53 0.03 4.26e-84
## scale(max_age_center_other, scale = FALSE)                0.02 0.00 1.40e-56
## I(scale(max_age_center_other, scale = FALSE)^2)           0.00 0.00 5.18e-11
## G_std:Sex                                                 0.01 0.02 6.66e-01
## G_std:scale(max_age_center_other, scale = FALSE)          0.00 0.00 4.61e-01
## Sex:scale(max_age_center_other, scale = FALSE)            0.01 0.00 3.62e-02
## G_std:I(scale(max_age_center_other, scale = FALSE)^2)     0.00 0.00 4.45e-01
## G_std:Sex:scale(max_age_center_other, scale = FALSE)      0.00 0.00 9.52e-02
##                                                         OR
## (Intercept)                                           0.02
## G_std                                                 1.09
## Sex                                                   1.70
## scale(max_age_center_other, scale = FALSE)            1.02
## I(scale(max_age_center_other, scale = FALSE)^2)       1.00
## G_std:Sex                                             1.01
## G_std:scale(max_age_center_other, scale = FALSE)      1.00
## Sex:scale(max_age_center_other, scale = FALSE)        1.01
## G_std:I(scale(max_age_center_other, scale = FALSE)^2) 1.00
## G_std:Sex:scale(max_age_center_other, scale = FALSE)  1.00
```

## 4. Probability of having a phenotype as a function of the g-factor

### a) Without data points

```
## Using data Phenotypes_Allergies_Other_0_3_no_na from global environment.
## This could cause incorrect results if Phenotypes_Allergies_Other_0_3_no_na
## has been altered since the model was fit. You can manually provide the data
## to the "data =" argument.
```

### b) With data points

```
## Using data Phenotypes_Allergies_Other_0_3_no_na from global environment.
## This could cause incorrect results if Phenotypes_Allergies_Other_0_3_no_na
## has been altered since the model was fit. You can manually provide the data
## to the "data =" argument.
```
