# Supplementary material for: High intelligence is not associated with a greater propensity for mental health disorders
Source: Eur Psychiatry. 2022 Nov 18;66(1):e3. doi: 10.1192/j.eurpsy.2022.2343 (PMC9879926; doi:10.1192/j.eurpsy.2022.2343)
Supplement: Supplementary file 1 [file S0924933822023434sup001.zip › S0924933822023434sup017.html]

Alcohol


# Alcohol

#### 2022-09-21

## 1. Regression with g-factor Group

```
## [1] "There are 123674 individuals without missing data in this analysis."
```

```
##                                                            Estimate   SE
## (Intercept)                                                   -2.86 0.02
## CA_GroupHigh_CA                                               -0.08 0.06
## CA_GroupLow_CA                                                -0.07 0.12
## Sex                                                           -0.90 0.03
## scale(max_age_alcohol, scale = FALSE)                         -0.04 0.00
## I(scale(max_age_alcohol, scale = FALSE)^2)                     0.00 0.00
## CA_GroupHigh_CA:Sex                                            0.23 0.10
## CA_GroupLow_CA:Sex                                            -0.10 0.18
## CA_GroupHigh_CA:scale(max_age_alcohol, scale = FALSE)          0.01 0.01
## CA_GroupLow_CA:scale(max_age_alcohol, scale = FALSE)           0.00 0.01
## Sex:scale(max_age_alcohol, scale = FALSE)                     -0.02 0.00
## CA_GroupHigh_CA:I(scale(max_age_alcohol, scale = FALSE)^2)     0.00 0.00
## CA_GroupLow_CA:I(scale(max_age_alcohol, scale = FALSE)^2)      0.00 0.00
## CA_GroupHigh_CA:Sex:scale(max_age_alcohol, scale = FALSE)      0.00 0.01
## CA_GroupLow_CA:Sex:scale(max_age_alcohol, scale = FALSE)      -0.03 0.02
##                                                                    p   OR
## (Intercept)                                                 0.00e+00 0.06
## CA_GroupHigh_CA                                             2.16e-01 0.92
## CA_GroupLow_CA                                              5.62e-01 0.93
## Sex                                                        9.97e-209 0.41
## scale(max_age_alcohol, scale = FALSE)                       1.38e-93 0.96
## I(scale(max_age_alcohol, scale = FALSE)^2)                  9.27e-08 1.00
## CA_GroupHigh_CA:Sex                                         1.94e-02 1.26
## CA_GroupLow_CA:Sex                                          5.92e-01 0.90
## CA_GroupHigh_CA:scale(max_age_alcohol, scale = FALSE)       1.44e-01 1.01
## CA_GroupLow_CA:scale(max_age_alcohol, scale = FALSE)        8.72e-01 1.00
## Sex:scale(max_age_alcohol, scale = FALSE)                   3.23e-06 0.98
## CA_GroupHigh_CA:I(scale(max_age_alcohol, scale = FALSE)^2)  2.90e-01 1.00
## CA_GroupLow_CA:I(scale(max_age_alcohol, scale = FALSE)^2)   7.93e-01 1.00
## CA_GroupHigh_CA:Sex:scale(max_age_alcohol, scale = FALSE)   9.13e-01 1.00
## CA_GroupLow_CA:Sex:scale(max_age_alcohol, scale = FALSE)    2.32e-01 0.97
```

## 2. Regression with g-factor Group Assumptions

### a) Influential values

There is no influential observations in our data.

```
## # A tibble: 3 × 12
##   Alcohol_Binary CA_Group   Sex `scale(max…`[,1] `I(scale(m…`[,1] .fitted .resid
##            <dbl> <fct>    <dbl>            <dbl>         <I<dbl>>   <dbl>  <dbl>
## 1              1 Low_CA    -0.5            -17.4             302.   -2.29   2.19
## 2              1 Low_CA     0.5             12.6             160.   -4.41   2.97
## 3              1 Low_CA    -0.5             14.8             219.   -2.96   2.45
## # … with 5 more variables: .std.resid <dbl>, .hat <dbl>, .sigma <dbl>,
## #   .cooksd <dbl>, index <int>
```

```
## # A tibble: 0 × 12
## # … with 12 variables: Alcohol_Binary <dbl>, CA_Group <fct>, Sex <dbl>,
## #   scale(max_age_alcohol, scale = FALSE) <dbl[,1]>,
## #   I(scale(max_age_alcohol, scale = FALSE)^2) <I<dbl[,1]>[,1]>, .fitted <dbl>,
## #   .resid <dbl>, .std.resid <dbl>, .hat <dbl>, .sigma <dbl>, .cooksd <dbl>,
## #   index <int>
```

```
##                                                                 Estimate
## (Intercept)                                                -2.8608863851
## CA_GroupHigh_CA                                            -0.0779471451
## CA_GroupLow_CA                                             -0.0685916193
## Sex                                                        -0.8979203733
## scale(max_age_alcohol, scale = FALSE)                      -0.0428576148
## I(scale(max_age_alcohol, scale = FALSE)^2)                 -0.0012174145
## CA_GroupHigh_CA:Sex                                         0.2321466873
## CA_GroupLow_CA:Sex                                         -0.0977094665
## CA_GroupHigh_CA:scale(max_age_alcohol, scale = FALSE)       0.0100950035
## CA_GroupLow_CA:scale(max_age_alcohol, scale = FALSE)       -0.0020076551
## Sex:scale(max_age_alcohol, scale = FALSE)                  -0.0171567730
## CA_GroupHigh_CA:I(scale(max_age_alcohol, scale = FALSE)^2)  0.0007511362
## CA_GroupLow_CA:I(scale(max_age_alcohol, scale = FALSE)^2)   0.0003581208
## CA_GroupHigh_CA:Sex:scale(max_age_alcohol, scale = FALSE)   0.0013496781
## CA_GroupLow_CA:Sex:scale(max_age_alcohol, scale = FALSE)   -0.0268877701
##                                                                      SE
## (Intercept)                                                0.0188907934
## CA_GroupHigh_CA                                            0.0629767794
## CA_GroupLow_CA                                             0.1182677144
## Sex                                                        0.0291236283
## scale(max_age_alcohol, scale = FALSE)                      0.0020884099
## I(scale(max_age_alcohol, scale = FALSE)^2)                 0.0002279574
## CA_GroupHigh_CA:Sex                                        0.0993087818
## CA_GroupLow_CA:Sex                                         0.1821370080
## CA_GroupHigh_CA:scale(max_age_alcohol, scale = FALSE)      0.0069019529
## CA_GroupLow_CA:scale(max_age_alcohol, scale = FALSE)       0.0124912739
## Sex:scale(max_age_alcohol, scale = FALSE)                  0.0036851805
## CA_GroupHigh_CA:I(scale(max_age_alcohol, scale = FALSE)^2) 0.0007101435
## CA_GroupLow_CA:I(scale(max_age_alcohol, scale = FALSE)^2)  0.0013679796
## CA_GroupHigh_CA:Sex:scale(max_age_alcohol, scale = FALSE)  0.0123238945
## CA_GroupLow_CA:Sex:scale(max_age_alcohol, scale = FALSE)   0.0225141930
##                                                                     t/z
## (Intercept)                                                -151.4434217
## CA_GroupHigh_CA                                              -1.2377125
## CA_GroupLow_CA                                               -0.5799691
## Sex                                                         -30.8313362
## scale(max_age_alcohol, scale = FALSE)                       -20.5216491
## I(scale(max_age_alcohol, scale = FALSE)^2)                   -5.3405357
## CA_GroupHigh_CA:Sex                                           2.3376250
## CA_GroupLow_CA:Sex                                           -0.5364614
## CA_GroupHigh_CA:scale(max_age_alcohol, scale = FALSE)         1.4626300
## CA_GroupLow_CA:scale(max_age_alcohol, scale = FALSE)         -0.1607246
## Sex:scale(max_age_alcohol, scale = FALSE)                    -4.6556127
## CA_GroupHigh_CA:I(scale(max_age_alcohol, scale = FALSE)^2)    1.0577246
## CA_GroupLow_CA:I(scale(max_age_alcohol, scale = FALSE)^2)     0.2617881
## CA_GroupHigh_CA:Sex:scale(max_age_alcohol, scale = FALSE)     0.1095172
## CA_GroupLow_CA:Sex:scale(max_age_alcohol, scale = FALSE)     -1.1942587
##                                                                        p
## (Intercept)                                                 0.000000e+00
## CA_GroupHigh_CA                                             2.158227e-01
## CA_GroupLow_CA                                              5.619355e-01
## Sex                                                        9.966880e-209
## scale(max_age_alcohol, scale = FALSE)                       1.379528e-93
## I(scale(max_age_alcohol, scale = FALSE)^2)                  9.267235e-08
## CA_GroupHigh_CA:Sex                                         1.940671e-02
## CA_GroupLow_CA:Sex                                          5.916397e-01
## CA_GroupHigh_CA:scale(max_age_alcohol, scale = FALSE)       1.435686e-01
## CA_GroupLow_CA:scale(max_age_alcohol, scale = FALSE)        8.723103e-01
## Sex:scale(max_age_alcohol, scale = FALSE)                   3.230187e-06
## CA_GroupHigh_CA:I(scale(max_age_alcohol, scale = FALSE)^2)  2.901810e-01
## CA_GroupLow_CA:I(scale(max_age_alcohol, scale = FALSE)^2)   7.934848e-01
## CA_GroupHigh_CA:Sex:scale(max_age_alcohol, scale = FALSE)   9.127923e-01
## CA_GroupLow_CA:Sex:scale(max_age_alcohol, scale = FALSE)    2.323768e-01
##                                                                         Model
## (Intercept)                                                Alcohol_Binary 0_3
## CA_GroupHigh_CA                                            Alcohol_Binary 0_3
## CA_GroupLow_CA                                             Alcohol_Binary 0_3
## Sex                                                        Alcohol_Binary 0_3
## scale(max_age_alcohol, scale = FALSE)                      Alcohol_Binary 0_3
## I(scale(max_age_alcohol, scale = FALSE)^2)                 Alcohol_Binary 0_3
## CA_GroupHigh_CA:Sex                                        Alcohol_Binary 0_3
## CA_GroupLow_CA:Sex                                         Alcohol_Binary 0_3
## CA_GroupHigh_CA:scale(max_age_alcohol, scale = FALSE)      Alcohol_Binary 0_3
## CA_GroupLow_CA:scale(max_age_alcohol, scale = FALSE)       Alcohol_Binary 0_3
## Sex:scale(max_age_alcohol, scale = FALSE)                  Alcohol_Binary 0_3
## CA_GroupHigh_CA:I(scale(max_age_alcohol, scale = FALSE)^2) Alcohol_Binary 0_3
## CA_GroupLow_CA:I(scale(max_age_alcohol, scale = FALSE)^2)  Alcohol_Binary 0_3
## CA_GroupHigh_CA:Sex:scale(max_age_alcohol, scale = FALSE)  Alcohol_Binary 0_3
## CA_GroupLow_CA:Sex:scale(max_age_alcohol, scale = FALSE)   Alcohol_Binary 0_3
```

### b) Multicollinearity

As a rule of thumb, a VIF value that exceeds 5 or 10 indicates a
problematic amount of collinearity.

```
## there are higher-order terms (interactions) in this model
## consider setting terms = 'marginal' or 'high-order'; see ?vif
```

```
##                                                         GVIF Df GVIF^(1/(2*Df))
## CA_Group                                            3.998696  2        1.414098
## Sex                                                 1.246564  1        1.116497
## scale(max_age_alcohol, scale = FALSE)               1.607185  1        1.267748
## I(scale(max_age_alcohol, scale = FALSE)^2)          1.492123  1        1.221525
## CA_Group:Sex                                        1.646573  2        1.132779
## CA_Group:scale(max_age_alcohol, scale = FALSE)      2.554410  2        1.264220
## Sex:scale(max_age_alcohol, scale = FALSE)           1.300809  1        1.140530
## CA_Group:I(scale(max_age_alcohol, scale = FALSE)^2) 6.091183  2        1.570997
## CA_Group:Sex:scale(max_age_alcohol, scale = FALSE)  1.659774  2        1.135043
```

## 3. Regression with g-factor

```
## [1] "There are 123674 individuals without missing data in this analysis."
```

```
##                                                  Estimate   SE         p   OR
## (Intercept)                                         -2.86 0.02  0.00e+00 0.06
## G_std                                               -0.02 0.01  1.74e-01 0.98
## Sex                                                 -0.90 0.03 1.30e-213 0.41
## scale(max_age_alcohol, scale = FALSE)               -0.04 0.00  9.74e-98 0.96
## I(scale(max_age_alcohol, scale = FALSE)^2)           0.00 0.00  1.66e-08 1.00
## G_std:Sex                                            0.05 0.02  1.70e-02 1.05
## G_std:scale(max_age_alcohol, scale = FALSE)          0.00 0.00  2.68e-02 1.00
## Sex:scale(max_age_alcohol, scale = FALSE)           -0.02 0.00  4.52e-07 0.98
## G_std:I(scale(max_age_alcohol, scale = FALSE)^2)     0.00 0.00  4.89e-02 1.00
## G_std:Sex:scale(max_age_alcohol, scale = FALSE)      0.00 0.00  4.39e-01 1.00
```

## 4. Probability of having a phenotype as a function of the g-factor

### a) Without data points

```
## Using data Alcohol_DF_items_0_3 from global environment. This could cause
## incorrect results if Alcohol_DF_items_0_3 has been altered since the model
## was fit. You can manually provide the data to the "data =" argument.
```

### b) With data points

```
## Using data Alcohol_DF_items_0_3 from global environment. This could cause
## incorrect results if Alcohol_DF_items_0_3 has been altered since the model
## was fit. You can manually provide the data to the "data =" argument.
```
