# Supplementary material for: High intelligence is not associated with a greater propensity for mental health disorders
Source: Eur Psychiatry. 2022 Nov 18;66(1):e3. doi: 10.1192/j.eurpsy.2022.2343 (PMC9879926; doi:10.1192/j.eurpsy.2022.2343)
Supplement: Supplementary file 1 [file S0924933822023434sup001.zip › S0924933822023434sup018.html]

Eczema


# Eczema

#### 2022-09-21

## 1. CA Group Regressions

```
age_sex_model_binary <- glm(Phenotype_Eczema_0_3 ~ CA_Group* Sex*scale(max_age_center_Eczema, scale = FALSE)  + 
                              CA_Group*Sex*I(scale((max_age_center_Eczema), scale = FALSE)^2) , data = Phenotypes_Allergies_Eczema_no_na, 
                            family = binomial)
Summary_age_sex_model_binary_Phenotype_HFR <- as.data.frame(summary(age_sex_model_binary)$coefficients)
Summary_age_sex_model_binary_Phenotype_HFR$Model <- "Eczema"
names(Summary_age_sex_model_binary_Phenotype_HFR) <- c("Estimate", "SE", "t/z", "p", "Model")
Summary_ChildA <- Summary_age_sex_model_binary_Phenotype_HFR
```

```
## [1] "There are 261395 individuals without missing data in this analysis."
```

```
##                                                                        Estimate
## (Intercept)                                                               -3.49
## CA_GroupHigh_CA                                                            0.22
## CA_GroupLow_CA                                                            -0.23
## Sex                                                                        0.03
## scale(max_age_center_Eczema, scale = FALSE)                                0.01
## I(scale((max_age_center_Eczema), scale = FALSE)^2)                         0.00
## CA_GroupHigh_CA:Sex                                                        0.09
## CA_GroupLow_CA:Sex                                                         0.10
## CA_GroupHigh_CA:scale(max_age_center_Eczema, scale = FALSE)                0.01
## CA_GroupLow_CA:scale(max_age_center_Eczema, scale = FALSE)                 0.00
## Sex:scale(max_age_center_Eczema, scale = FALSE)                           -0.01
## CA_GroupHigh_CA:I(scale((max_age_center_Eczema), scale = FALSE)^2)         0.00
## CA_GroupLow_CA:I(scale((max_age_center_Eczema), scale = FALSE)^2)          0.00
## Sex:I(scale((max_age_center_Eczema), scale = FALSE)^2)                     0.00
## CA_GroupHigh_CA:Sex:scale(max_age_center_Eczema, scale = FALSE)            0.01
## CA_GroupLow_CA:Sex:scale(max_age_center_Eczema, scale = FALSE)            -0.01
## CA_GroupHigh_CA:Sex:I(scale((max_age_center_Eczema), scale = FALSE)^2)     0.00
## CA_GroupLow_CA:Sex:I(scale((max_age_center_Eczema), scale = FALSE)^2)      0.00
##                                                                          SE
## (Intercept)                                                            0.02
## CA_GroupHigh_CA                                                        0.06
## CA_GroupLow_CA                                                         0.09
## Sex                                                                    0.03
## scale(max_age_center_Eczema, scale = FALSE)                            0.00
## I(scale((max_age_center_Eczema), scale = FALSE)^2)                     0.00
## CA_GroupHigh_CA:Sex                                                    0.11
## CA_GroupLow_CA:Sex                                                     0.19
## CA_GroupHigh_CA:scale(max_age_center_Eczema, scale = FALSE)            0.00
## CA_GroupLow_CA:scale(max_age_center_Eczema, scale = FALSE)             0.01
## Sex:scale(max_age_center_Eczema, scale = FALSE)                        0.00
## CA_GroupHigh_CA:I(scale((max_age_center_Eczema), scale = FALSE)^2)     0.00
## CA_GroupLow_CA:I(scale((max_age_center_Eczema), scale = FALSE)^2)      0.00
## Sex:I(scale((max_age_center_Eczema), scale = FALSE)^2)                 0.00
## CA_GroupHigh_CA:Sex:scale(max_age_center_Eczema, scale = FALSE)        0.01
## CA_GroupLow_CA:Sex:scale(max_age_center_Eczema, scale = FALSE)         0.02
## CA_GroupHigh_CA:Sex:I(scale((max_age_center_Eczema), scale = FALSE)^2) 0.00
## CA_GroupLow_CA:Sex:I(scale((max_age_center_Eczema), scale = FALSE)^2)  0.00
##                                                                               p
## (Intercept)                                                            0.00e+00
## CA_GroupHigh_CA                                                        7.61e-05
## CA_GroupLow_CA                                                         1.64e-02
## Sex                                                                    3.82e-01
## scale(max_age_center_Eczema, scale = FALSE)                            9.22e-11
## I(scale((max_age_center_Eczema), scale = FALSE)^2)                     3.16e-15
## CA_GroupHigh_CA:Sex                                                    4.01e-01
## CA_GroupLow_CA:Sex                                                     6.05e-01
## CA_GroupHigh_CA:scale(max_age_center_Eczema, scale = FALSE)            1.62e-01
## CA_GroupLow_CA:scale(max_age_center_Eczema, scale = FALSE)             9.82e-01
## Sex:scale(max_age_center_Eczema, scale = FALSE)                        3.63e-03
## CA_GroupHigh_CA:I(scale((max_age_center_Eczema), scale = FALSE)^2)     5.15e-01
## CA_GroupLow_CA:I(scale((max_age_center_Eczema), scale = FALSE)^2)      7.45e-01
## Sex:I(scale((max_age_center_Eczema), scale = FALSE)^2)                 3.46e-02
## CA_GroupHigh_CA:Sex:scale(max_age_center_Eczema, scale = FALSE)        3.61e-01
## CA_GroupLow_CA:Sex:scale(max_age_center_Eczema, scale = FALSE)         6.08e-01
## CA_GroupHigh_CA:Sex:I(scale((max_age_center_Eczema), scale = FALSE)^2) 8.48e-01
## CA_GroupLow_CA:Sex:I(scale((max_age_center_Eczema), scale = FALSE)^2)  5.61e-01
##                                                                          OR
## (Intercept)                                                            0.03
## CA_GroupHigh_CA                                                        1.25
## CA_GroupLow_CA                                                         0.79
## Sex                                                                    1.03
## scale(max_age_center_Eczema, scale = FALSE)                            1.01
## I(scale((max_age_center_Eczema), scale = FALSE)^2)                     1.00
## CA_GroupHigh_CA:Sex                                                    1.09
## CA_GroupLow_CA:Sex                                                     1.11
## CA_GroupHigh_CA:scale(max_age_center_Eczema, scale = FALSE)            1.01
## CA_GroupLow_CA:scale(max_age_center_Eczema, scale = FALSE)             1.00
## Sex:scale(max_age_center_Eczema, scale = FALSE)                        0.99
## CA_GroupHigh_CA:I(scale((max_age_center_Eczema), scale = FALSE)^2)     1.00
## CA_GroupLow_CA:I(scale((max_age_center_Eczema), scale = FALSE)^2)      1.00
## Sex:I(scale((max_age_center_Eczema), scale = FALSE)^2)                 1.00
## CA_GroupHigh_CA:Sex:scale(max_age_center_Eczema, scale = FALSE)        1.01
## CA_GroupLow_CA:Sex:scale(max_age_center_Eczema, scale = FALSE)         0.99
## CA_GroupHigh_CA:Sex:I(scale((max_age_center_Eczema), scale = FALSE)^2) 1.00
## CA_GroupLow_CA:Sex:I(scale((max_age_center_Eczema), scale = FALSE)^2)  1.00
```

## 2. Regression with g-factor Group Assumptions

### a) Influential values

There is no influential observations in our data.

```
## # A tibble: 3 × 12
##   Phenotype_Eczema_0_3 CA_Group   Sex `scale(max_…`[,1] `I(scale((…`[,1] .fitted
##                  <dbl> <fct>    <dbl>             <dbl>         <I<dbl>>   <dbl>
## 1                    1 Low_CA    -0.5              20.6             424.   -3.16
## 2                    1 Low_CA    -0.5              21.0             441.   -3.14
## 3                    1 Low_CA    -0.5              18.6             345.   -3.25
## # … with 6 more variables: .resid <dbl>, .std.resid <dbl>, .hat <dbl>,
## #   .sigma <dbl>, .cooksd <dbl>, index <int>
```

```
## # A tibble: 0 × 12
## # … with 12 variables: Phenotype_Eczema_0_3 <dbl>, CA_Group <fct>, Sex <dbl>,
## #   scale(max_age_center_Eczema, scale = FALSE) <dbl[,1]>,
## #   I(scale((max_age_center_Eczema), scale = FALSE)^2) <I<dbl[,1]>[,1]>,
## #   .fitted <dbl>, .resid <dbl>, .std.resid <dbl>, .hat <dbl>, .sigma <dbl>,
## #   .cooksd <dbl>, index <int>
```

### b) Multicollinearity

As a rule of thumb, a VIF value that exceeds 5 or 10 indicates a
problematic amount of collinearity.

```
## there are higher-order terms (interactions) in this model
## consider setting terms = 'marginal' or 'high-order'; see ?vif
```

```
##                                                                     GVIF Df
## CA_Group                                                        3.514808  2
## Sex                                                             2.056148  1
## scale(max_age_center_Eczema, scale = FALSE)                     1.172918  1
## I(scale((max_age_center_Eczema), scale = FALSE)^2)              1.184727  1
## CA_Group:Sex                                                    3.919719  2
## CA_Group:scale(max_age_center_Eczema, scale = FALSE)            1.272159  2
## Sex:scale(max_age_center_Eczema, scale = FALSE)                 1.168528  1
## CA_Group:I(scale((max_age_center_Eczema), scale = FALSE)^2)     3.963048  2
## Sex:I(scale((max_age_center_Eczema), scale = FALSE)^2)          2.121075  1
## CA_Group:Sex:scale(max_age_center_Eczema, scale = FALSE)        1.272236  2
## CA_Group:Sex:I(scale((max_age_center_Eczema), scale = FALSE)^2) 4.147600  2
##                                                                 GVIF^(1/(2*Df))
## CA_Group                                                               1.369227
## Sex                                                                    1.433928
## scale(max_age_center_Eczema, scale = FALSE)                            1.083014
## I(scale((max_age_center_Eczema), scale = FALSE)^2)                     1.088452
## CA_Group:Sex                                                           1.407064
## CA_Group:scale(max_age_center_Eczema, scale = FALSE)                   1.062026
## Sex:scale(max_age_center_Eczema, scale = FALSE)                        1.080985
## CA_Group:I(scale((max_age_center_Eczema), scale = FALSE)^2)            1.410936
## Sex:I(scale((max_age_center_Eczema), scale = FALSE)^2)                 1.456391
## CA_Group:Sex:scale(max_age_center_Eczema, scale = FALSE)               1.062043
## CA_Group:Sex:I(scale((max_age_center_Eczema), scale = FALSE)^2)        1.427083
```

## 3. Regression with g-factor

```
## [1] "There are 261395 individuals without missing data in this analysis."
```

```
##                                                        Estimate   SE        p
## (Intercept)                                               -3.51 0.02 0.00e+00
## G_std                                                      0.09 0.01 4.24e-14
## Sex                                                        0.01 0.02 7.83e-01
## scale(max_age_center_Eczema, scale = FALSE)                0.01 0.00 1.39e-14
## I(scale(max_age_center_Eczema, scale = FALSE)^2)           0.00 0.00 1.33e-16
## G_std:Sex                                                  0.00 0.02 8.96e-01
## G_std:scale(max_age_center_Eczema, scale = FALSE)          0.00 0.00 3.34e-01
## Sex:scale(max_age_center_Eczema, scale = FALSE)           -0.01 0.00 2.46e-03
## G_std:I(scale(max_age_center_Eczema, scale = FALSE)^2)     0.00 0.00 6.62e-01
## G_std:Sex:scale(max_age_center_Eczema, scale = FALSE)      0.00 0.00 1.45e-01
##                                                          OR
## (Intercept)                                            0.03
## G_std                                                  1.09
## Sex                                                    1.01
## scale(max_age_center_Eczema, scale = FALSE)            1.01
## I(scale(max_age_center_Eczema, scale = FALSE)^2)       1.00
## G_std:Sex                                              1.00
## G_std:scale(max_age_center_Eczema, scale = FALSE)      1.00
## Sex:scale(max_age_center_Eczema, scale = FALSE)        0.99
## G_std:I(scale(max_age_center_Eczema, scale = FALSE)^2) 1.00
## G_std:Sex:scale(max_age_center_Eczema, scale = FALSE)  1.00
```

## 4. Probability of having a phenotype as a function of the g-factor

### a) Without data points

```
## Using data Phenotypes_Allergies_Eczema_no_na from global environment. This
## could cause incorrect results if Phenotypes_Allergies_Eczema_no_na has been
## altered since the model was fit. You can manually provide the data to the
## "data =" argument.
```

### b) With data points

```
## Using data Phenotypes_Allergies_Eczema_no_na from global environment. This
## could cause incorrect results if Phenotypes_Allergies_Eczema_no_na has been
## altered since the model was fit. You can manually provide the data to the
## "data =" argument.
```
