# Supplementary material for: High intelligence is not associated with a greater propensity for mental health disorders
Source: Eur Psychiatry. 2022 Nov 18;66(1):e3. doi: 10.1192/j.eurpsy.2022.2343 (PMC9879926; doi:10.1192/j.eurpsy.2022.2343)
Supplement: Supplementary file 1 [file S0924933822023434sup001.zip › S0924933822023434sup019.html]

General Allergy


# General Allergy

#### 2022-09-21

## 1. Regression with g-factor Group

```
## [1] "There are 102837 individuals without missing data in this analysis."
```

```
##                                                                          Estimate
## (Intercept)                                                                 -0.92
## CA_GroupHigh_CA                                                              0.12
## CA_GroupLow_CA                                                              -0.17
## Sex                                                                          0.10
## scale(max_age_general_allergy, scale = FALSE)                               -0.02
## I(scale((max_age_general_allergy), scale = FALSE)^2)                         0.00
## CA_GroupHigh_CA:Sex                                                          0.06
## CA_GroupLow_CA:Sex                                                          -0.01
## CA_GroupHigh_CA:scale(max_age_general_allergy, scale = FALSE)                0.01
## CA_GroupLow_CA:scale(max_age_general_allergy, scale = FALSE)                 0.01
## CA_GroupHigh_CA:I(scale((max_age_general_allergy), scale = FALSE)^2)         0.00
## CA_GroupLow_CA:I(scale((max_age_general_allergy), scale = FALSE)^2)          0.00
## Sex:I(scale((max_age_general_allergy), scale = FALSE)^2)                     0.00
## CA_GroupHigh_CA:Sex:I(scale((max_age_general_allergy), scale = FALSE)^2)     0.00
## CA_GroupLow_CA:Sex:I(scale((max_age_general_allergy), scale = FALSE)^2)      0.00
##                                                                            SE
## (Intercept)                                                              0.01
## CA_GroupHigh_CA                                                          0.03
## CA_GroupLow_CA                                                           0.07
## Sex                                                                      0.02
## scale(max_age_general_allergy, scale = FALSE)                            0.00
## I(scale((max_age_general_allergy), scale = FALSE)^2)                     0.00
## CA_GroupHigh_CA:Sex                                                      0.06
## CA_GroupLow_CA:Sex                                                       0.14
## CA_GroupHigh_CA:scale(max_age_general_allergy, scale = FALSE)            0.00
## CA_GroupLow_CA:scale(max_age_general_allergy, scale = FALSE)             0.01
## CA_GroupHigh_CA:I(scale((max_age_general_allergy), scale = FALSE)^2)     0.00
## CA_GroupLow_CA:I(scale((max_age_general_allergy), scale = FALSE)^2)      0.00
## Sex:I(scale((max_age_general_allergy), scale = FALSE)^2)                 0.00
## CA_GroupHigh_CA:Sex:I(scale((max_age_general_allergy), scale = FALSE)^2) 0.00
## CA_GroupLow_CA:Sex:I(scale((max_age_general_allergy), scale = FALSE)^2)  0.00
##                                                                                  p
## (Intercept)                                                               0.00e+00
## CA_GroupHigh_CA                                                           6.29e-05
## CA_GroupLow_CA                                                            1.51e-02
## Sex                                                                       1.47e-06
## scale(max_age_general_allergy, scale = FALSE)                            4.81e-115
## I(scale((max_age_general_allergy), scale = FALSE)^2)                      3.19e-02
## CA_GroupHigh_CA:Sex                                                       3.49e-01
## CA_GroupLow_CA:Sex                                                        9.68e-01
## CA_GroupHigh_CA:scale(max_age_general_allergy, scale = FALSE)             2.43e-02
## CA_GroupLow_CA:scale(max_age_general_allergy, scale = FALSE)              3.96e-01
## CA_GroupHigh_CA:I(scale((max_age_general_allergy), scale = FALSE)^2)      2.63e-01
## CA_GroupLow_CA:I(scale((max_age_general_allergy), scale = FALSE)^2)       5.13e-01
## Sex:I(scale((max_age_general_allergy), scale = FALSE)^2)                  2.60e-01
## CA_GroupHigh_CA:Sex:I(scale((max_age_general_allergy), scale = FALSE)^2)  9.68e-02
## CA_GroupLow_CA:Sex:I(scale((max_age_general_allergy), scale = FALSE)^2)   9.51e-01
##                                                                            OR
## (Intercept)                                                              0.40
## CA_GroupHigh_CA                                                          1.13
## CA_GroupLow_CA                                                           0.84
## Sex                                                                      1.11
## scale(max_age_general_allergy, scale = FALSE)                            0.98
## I(scale((max_age_general_allergy), scale = FALSE)^2)                     1.00
## CA_GroupHigh_CA:Sex                                                      1.06
## CA_GroupLow_CA:Sex                                                       0.99
## CA_GroupHigh_CA:scale(max_age_general_allergy, scale = FALSE)            1.01
## CA_GroupLow_CA:scale(max_age_general_allergy, scale = FALSE)             1.01
## CA_GroupHigh_CA:I(scale((max_age_general_allergy), scale = FALSE)^2)     1.00
## CA_GroupLow_CA:I(scale((max_age_general_allergy), scale = FALSE)^2)      1.00
## Sex:I(scale((max_age_general_allergy), scale = FALSE)^2)                 1.00
## CA_GroupHigh_CA:Sex:I(scale((max_age_general_allergy), scale = FALSE)^2) 1.00
## CA_GroupLow_CA:Sex:I(scale((max_age_general_allergy), scale = FALSE)^2)  1.00
```

## 2. Regression with g-factor Group Assumptions

### a) Influential values

There is no influential observations in our data.

```
## # A tibble: 3 × 12
##   General_Allergy_0_3 CA_Group   Sex `scale(max_a…`[,1] `I(scale((…`[,1] .fitted
##                 <dbl> <fct>    <dbl>              <dbl>         <I<dbl>>   <dbl>
## 1                   1 Low_CA    -0.5              -17.7             314.  -0.675
## 2                   1 Low_CA     0.5              -17.8             317.  -0.692
## 3                   1 Low_CA    -0.5               16.6             276.  -1.31 
## # … with 6 more variables: .resid <dbl>, .std.resid <dbl>, .hat <dbl>,
## #   .sigma <dbl>, .cooksd <dbl>, index <int>
```

```
## # A tibble: 0 × 12
## # … with 12 variables: General_Allergy_0_3 <dbl>, CA_Group <fct>, Sex <dbl>,
## #   scale(max_age_general_allergy, scale = FALSE) <dbl[,1]>,
## #   I(scale((max_age_general_allergy), scale = FALSE)^2) <I<dbl[,1]>[,1]>,
## #   .fitted <dbl>, .resid <dbl>, .std.resid <dbl>, .hat <dbl>, .sigma <dbl>,
## #   .cooksd <dbl>, index <int>
```

### b) Multicollinearity

As a rule of thumb, a VIF value that exceeds 5 or 10 indicates a
problematic amount of collinearity.

```
## there are higher-order terms (interactions) in this model
## consider setting terms = 'marginal' or 'high-order'; see ?vif
```

```
##                                                                       GVIF Df
## CA_Group                                                          3.763101  2
## Sex                                                               2.100360  1
## scale(max_age_general_allergy, scale = FALSE)                     1.330532  1
## I(scale((max_age_general_allergy), scale = FALSE)^2)              1.348651  1
## CA_Group:Sex                                                      4.001162  2
## CA_Group:scale(max_age_general_allergy, scale = FALSE)            1.471575  2
## CA_Group:I(scale((max_age_general_allergy), scale = FALSE)^2)     4.638974  2
## Sex:I(scale((max_age_general_allergy), scale = FALSE)^2)          2.138713  1
## CA_Group:Sex:I(scale((max_age_general_allergy), scale = FALSE)^2) 3.909847  2
##                                                                   GVIF^(1/(2*Df))
## CA_Group                                                                 1.392793
## Sex                                                                      1.449262
## scale(max_age_general_allergy, scale = FALSE)                            1.153487
## I(scale((max_age_general_allergy), scale = FALSE)^2)                     1.161314
## CA_Group:Sex                                                             1.414316
## CA_Group:scale(max_age_general_allergy, scale = FALSE)                   1.101401
## CA_Group:I(scale((max_age_general_allergy), scale = FALSE)^2)            1.467592
## Sex:I(scale((max_age_general_allergy), scale = FALSE)^2)                 1.462434
## CA_Group:Sex:I(scale((max_age_general_allergy), scale = FALSE)^2)        1.406177
```

## 3. Regression with g-factor

```
## [1] "There are 102837 individuals without missing data in this analysis."
```

```
##                                                          Estimate   SE
## (Intercept)                                                 -0.93 0.01
## G_std                                                        0.06 0.01
## Sex                                                          0.09 0.02
## scale(max_age_general_allergy, scale = FALSE)               -0.02 0.00
## I(scale(max_age_general_allergy, scale = FALSE)^2)           0.00 0.00
## G_std:Sex                                                   -0.01 0.01
## G_std:scale(max_age_general_allergy, scale = FALSE)          0.00 0.00
## Sex:scale(max_age_general_allergy, scale = FALSE)            0.00 0.00
## G_std:I(scale(max_age_general_allergy, scale = FALSE)^2)     0.00 0.00
## G_std:Sex:scale(max_age_general_allergy, scale = FALSE)      0.00 0.00
##                                                                  p   OR
## (Intercept)                                               0.00e+00 0.39
## G_std                                                     6.30e-13 1.06
## Sex                                                       1.00e-09 1.09
## scale(max_age_general_allergy, scale = FALSE)            4.23e-108 0.98
## I(scale(max_age_general_allergy, scale = FALSE)^2)        8.17e-02 1.00
## G_std:Sex                                                 2.69e-01 0.99
## G_std:scale(max_age_general_allergy, scale = FALSE)       3.69e-01 1.00
## Sex:scale(max_age_general_allergy, scale = FALSE)         7.37e-02 1.00
## G_std:I(scale(max_age_general_allergy, scale = FALSE)^2)  7.92e-01 1.00
## G_std:Sex:scale(max_age_general_allergy, scale = FALSE)   1.42e-01 1.00
```

## 4. Probability of having a phenotype as a function of the g-factor

### a) Without data points

```
## Using data Phenotypes_Allergies_Final_0_3 from global environment. This
## could cause incorrect results if Phenotypes_Allergies_Final_0_3 has been
## altered since the model was fit. You can manually provide the data to the
## "data =" argument.
```

### b) With data points

```
## Using data Phenotypes_Allergies_Final_0_3 from global environment. This
## could cause incorrect results if Phenotypes_Allergies_Final_0_3 has been
## altered since the model was fit. You can manually provide the data to the
## "data =" argument.
```
