# Supplementary material for: High intelligence is not associated with a greater propensity for mental health disorders
Source: Eur Psychiatry. 2022 Nov 18;66(1):e3. doi: 10.1192/j.eurpsy.2022.2343 (PMC9879926; doi:10.1192/j.eurpsy.2022.2343)
Supplement: Supplementary file 1 [file S0924933822023434sup001.zip › S0924933822023434sup020.html]

Asthma


# Asthma

#### 2022-09-21

## 1. Regression with g-factor Group

```
## [1] "There are 102837 individuals without missing data in this analysis."
```

```
##                                                    Estimate   SE        p   OR
## (Intercept)                                           -1.73 0.01 0.00e+00 0.18
## CA_GroupHigh_CA                                       -0.04 0.04 3.61e-01 0.96
## CA_GroupLow_CA                                         0.16 0.08 4.70e-02 1.17
## Sex                                                    0.09 0.02 9.69e-07 1.09
## scale(max_age, scale = FALSE)                         -0.02 0.00 2.30e-34 0.98
## I(scale(max_age, scale = FALSE)^2)                     0.00 0.00 3.01e-02 1.00
## CA_GroupHigh_CA:Sex                                    0.02 0.06 7.64e-01 1.02
## CA_GroupLow_CA:Sex                                     0.11 0.12 3.35e-01 1.12
## CA_GroupHigh_CA:scale(max_age, scale = FALSE)          0.01 0.00 1.64e-01 1.01
## CA_GroupLow_CA:scale(max_age, scale = FALSE)           0.00 0.01 9.03e-01 1.00
## Sex:scale(max_age, scale = FALSE)                      0.00 0.00 1.06e-01 1.00
## CA_GroupHigh_CA:I(scale(max_age, scale = FALSE)^2)     0.00 0.00 7.03e-01 1.00
## CA_GroupLow_CA:I(scale(max_age, scale = FALSE)^2)      0.00 0.00 5.04e-01 1.00
## CA_GroupHigh_CA:Sex:scale(max_age, scale = FALSE)      0.00 0.01 7.73e-01 1.00
## CA_GroupLow_CA:Sex:scale(max_age, scale = FALSE)       0.01 0.02 3.48e-01 1.01
```

## 2. Regression with g-factor Group Assumptions

### a) Influential values

There is no influential observations in our data.

```
## # A tibble: 3 × 12
##   Asthma_Binary CA_Group   Sex `scale(max_…`[,1] `I(scale(m…`[,1] .fitted .resid
##           <dbl> <fct>    <dbl>             <dbl>         <I<dbl>>   <dbl>  <dbl>
## 1             1 Low_CA    -0.5             -16.7             277.   -1.33   1.77
## 2             1 Low_CA    -0.5              13.7             187.   -2.08   2.10
## 3             1 Low_CA     0.5             -17.5             306.   -1.43   1.81
## # … with 5 more variables: .std.resid <dbl>, .hat <dbl>, .sigma <dbl>,
## #   .cooksd <dbl>, index <int>
```

```
## # A tibble: 0 × 12
## # … with 12 variables: Asthma_Binary <dbl>, CA_Group <fct>, Sex <dbl>,
## #   scale(max_age, scale = FALSE) <dbl[,1]>,
## #   I(scale(max_age, scale = FALSE)^2) <I<dbl[,1]>[,1]>, .fitted <dbl>,
## #   .resid <dbl>, .std.resid <dbl>, .hat <dbl>, .sigma <dbl>, .cooksd <dbl>,
## #   index <int>
```

### b) Multicollinearity

As a rule of thumb, a VIF value that exceeds 5 or 10 indicates a
problematic amount of collinearity.

```
## there are higher-order terms (interactions) in this model
## consider setting terms = 'marginal' or 'high-order'; see ?vif
```

```
##                                                 GVIF Df GVIF^(1/(2*Df))
## CA_Group                                    3.739348  2        1.390590
## Sex                                         1.155282  1        1.074840
## scale(max_age, scale = FALSE)               1.342328  1        1.158589
## I(scale(max_age, scale = FALSE)^2)          1.334435  1        1.155178
## CA_Group:Sex                                1.245234  2        1.056362
## CA_Group:scale(max_age, scale = FALSE)      1.543402  2        1.114602
## Sex:scale(max_age, scale = FALSE)           1.170066  1        1.081696
## CA_Group:I(scale(max_age, scale = FALSE)^2) 4.629894  2        1.466874
## CA_Group:Sex:scale(max_age, scale = FALSE)  1.207079  2        1.048175
```

## 3. Regression with g-factor

```
## [1] "There are 102837 individuals without missing data in this analysis."
```

```
##                                          Estimate   SE        p   OR
## (Intercept)                                 -1.73 0.01 0.00e+00 0.18
## G_std                                        0.00 0.01 6.83e-01 1.00
## Sex                                          0.10 0.02 9.88e-08 1.11
## scale(max_age, scale = FALSE)               -0.01 0.00 3.99e-31 0.99
## I(scale(max_age, scale = FALSE)^2)           0.00 0.00 1.22e-02 1.00
## G_std:Sex                                   -0.01 0.01 4.19e-01 0.99
## G_std:scale(max_age, scale = FALSE)          0.00 0.00 2.31e-01 1.00
## Sex:scale(max_age, scale = FALSE)            0.00 0.00 6.19e-02 1.00
## G_std:I(scale(max_age, scale = FALSE)^2)     0.00 0.00 2.75e-01 1.00
## G_std:Sex:scale(max_age, scale = FALSE)      0.00 0.00 2.69e-01 1.00
```

## 4. Probability of having a phenotype as a function of the g-factor

### a) Without data points

```
## Using data Asthma_DF_items_0_3 from global environment. This could cause
## incorrect results if Asthma_DF_items_0_3 has been altered since the model
## was fit. You can manually provide the data to the "data =" argument.
```

### b) With data points

```
## Using data Asthma_DF_items_0_3 from global environment. This could cause
## incorrect results if Asthma_DF_items_0_3 has been altered since the model
## was fit. You can manually provide the data to the "data =" argument.
```
